# Food safety and hygiene practices in the preparation of chicken meat and tomatoes in Harar, Ethiopia: Development of a household food safety index

**DOI:** 10.1016/j.onehlt.2026.101530

**Published:** 2026-07-28

**Authors:** Meseret Bekele, Kebede Amenu, Biruk Alemu Gemeda, Johanna F. Lindahl, Mohammed Ahmed, Sisay Girma, Theodore J.D. Knight-Jones, Delia Grace

**Affiliations:** a International Livestock Research Institute (ILRI), P.O. Box 5689, Addis Ababa, Ethiopia; b Global Health and Migration Unit, Department of Women's and Children's Health, Uppsala University, Akademiska sjukhuset, 751 85 Uppsala, Sweden; c Department of Animal Health and Antibiotic Strategies, Swedish Veterinary Agency, SE-751 89 Uppsala, Sweden; d Department of Veterinary Biosciences, University of Helsinki, 00014 Helsinki, Finland; e College of Veterinary Medicine, Haramaya University, P.O. Box 138, Dire Dawa, Ethiopia; f Natural Resources Institute, University of Greenwich, Central Avenue, Chatham Maritime, Kent ME4 4TB, UK; g Health Program, International Livestock Research Institute, Box, 30709 Nairobi, Kenya

**Keywords:** Chicken, Food safety, Households, Hygienic practice, One health, Ethiopia

## Abstract

Foodborne diseases are a critical One Health challenge, with household food handling practices and environmental hygiene being responsible for the transmission of foodborne pathogens. This study assessed household food hygiene practices during chicken and tomato meal preparation in Harar, Ethiopia. A cross-sectional survey was conducted among 252 urban households. Several high-risk practices were identified, including inadequate separation of raw and cooked foods; notably 74% of households used the same cutting board for meat and vegetables, and 35% reported inconsistent surface cleaning. A Household Good Food Hygienic Practices (HGFHP) index comprising 39 variables was developed as a measure of food safety behaviour. Households with good hygienic practices tended to have good neighbourhood infrastructure, access to food safety information from television. Unexpectedly, having no formal education was associated with better hygiene scores, as was being unconcerned about home slaughter as a risky practice. Findings revealed persistence of traditional food practices alongside modernization, with universal access to televisions and clean water. Television emerged as a key source of food safety information. Strong correlation between hygiene scores for chicken and tomatoes suggests common behavioural pathways affecting food safety across both food types. Targeted interventions, including mass media education and infrastructure improvements, are needed to improve household food safety and reduce the large and neglected burden of foodborne disease in Africa.

## Highlights

• Risky practices: 74% shared cutting boards for meat/vegetables, 35% poor cleaning.
• Hygiene improves with infrastructure, TV food information slaughter attitude.
• Unexpectedly, having no education correlated positively with better hygiene scores.
• Commodity-specific interventions have cross-commodity spillover effects.
• Hygiene is influenced more by infrastructure, information seeking, attitudes than education.

## 1 Introduction

Foodborne disease (FBD) is a major public health challenge and an important One Health issue, with the transmission of zoonotic pathogen strongly influenced by both environmental conditions and food preparation practices. In Africa, FBD is estimated to cause approximately 160 million illnesses and 210,000 deaths in 2023 [1]. The productivity losses for Sub-Saharan Africa measured in lost human capital are estimated at US$16.5 billion per annum including costs of illness and trade losses [2]. Recent studies found the economic cost of FBD associated with chicken meat and tomatoes in Ethiopia alone was USD192 million and USD 21 million, respectively in 2017, together amounting to 1% of the gross national income [3]. Although chicken is consumed much less frequently than tomatoes in Ethiopia [4], the burden associated with three important pathogens (*Campylobacter* spp., non-typhoidal *Salmonella enterica* and enterotoxigenic *Escherichia coli*) was estimated to be substantially higher for chicken than tomatoes partly due to cross-contamination. Together, these two foods contribute about 29% of the total burden of FBD for these pathogens [3].

Ethiopia, the second most populous country in Africa, has one of the fastest-growing economies in the region [5]. However, like other low- and middle-income countries (LMICs), ensuring food safety remains challenging due to several factors including inadequate infrastructure, lack of producer and consumer awareness, and limited implementation of regulations [2]. A recent study of tomato vendors in two Ethiopian cities (Harar and Dire Dawa) found that hygiene perception and practices were inadequate and infrastructure extremely deficient [6]. Related studies in Ethiopia and Burkina Faso also reported poor hygiene practices among chicken slaughterers and vendors [4], [6].

Animal source food (ASF) and fresh fruits and vegetables are the foods most often implicated as causes of FBD [7]. Poultry are recognized reservoirs of important zoonotic foodborne pathogens, including *Campylobacter* spp. and *Salmonella* spp., while household food preparation environments provide opportunities for pathogen transfer between raw foods, food-contact surfaces, and consumers. Consequently, food safety represents a critical One Health challenge requiring integrated approaches that address animal, human, and environmental health simultaneously [8]. A systematic literature review found widespread contamination of chicken, and fruit and vegetables in Ethiopia [9]. Chicken is an important source of meat in the country, typically eaten as a traditionally prepared, well-cooked chicken stew (*Doro wot*) on special occasions like holidays and social ceremonies. Demand for poultry is increasing rapidly, driven by population growth, urbanisation, increased incomes, and changing dietary habits, including adoption of new food preparation methods, such as roasting. Although backyard production remains the dominant supply system, a more commercialized poultry industry is emerging to meet the growing demand in the country [10]. The shortfall between demand and supply is met by significant importation of chicken meat [11]. A substantial share of poultry meat consumed in urban areas is imported, primarily from Brazil and, to a lesser extent, from European Union countries [12], [13].

While contamination can occur at any point along the value chain, household food handlers are regarded as the last and most critical ‘line of defence’ in preventing foodborne illness [14]. Household hygiene is particularly important in settings where slaughtering and carcases processing occur at home, as is common for chickens in African [15]. Despite its importance, household food handling and hygiene practices remain relatively understudied [14], especially in Ethiopia, where most previous studies have focused on production, processing or retail stages [9]. Evidence from other regions reinforces the importance of household practices in FBD prevention. For example, a study in Lebanon demonstrated that inadequate separation of raw and ready-to-eat foods, poor hand hygiene, and improper food storage were key contributors to contamination risks at the household level [16]. Similar findings from diverse settings highlight that cross-contamination from poor hygiene behaviour, and environmental conditions are central determinants of food safety. These patterns underscore the universal importance of household food handling practices in preventing FBD and highlight the need for context-specific interventions that address common underlying behavioural and infrastructural challenges.

This study aimed to assess household food handlers' food hygiene and safety practices during the preparation of chicken and vegetable (tomato) meals at household level, focusing on food hygiene and safety in Harar, Ethiopia, and identified factors associated with good food hygiene practices using novel index to evaluate household food safety situation.

## 2 Methods and materials

### 2.1 Project context

The study was carried out as part of the Pull-Push project^1^, which developed and evaluated context-specific food safety interventions in Ethiopia and Burkina Faso, aiming to improve food safety in urban informal markets by testing whether consumer demand for safe food can be enhanced and utilized to drive improved food safety standards, combining ‘pull’ approaches (consumer demand) with ‘push’ approaches (capacity building of market actors and regulators) to achieve sustainably improved food safety outcomes [17]. The project focused on the high-risk foods chicken and vegetables, specifically tomatoes. We focused on tomatoes for several reasons: (1) they are the most commonly consumed vegetable in urban Ethiopian diets, (2) they are typically consumed raw in salads, without the pathogen reduction step provided by cooking [4], (3) previous studies have documented food safety concerns in Ethiopian tomato value chains [6], (4) tomatoes are often prepared on the same surfaces as raw meat, creating cross-contamination risks, and (5) they are mostly cultivated by small-scale farmers using traditional farming methods and sold through largely unregulated informal value chains [18].

### 2.2 Study area

The study was conducted in Harar, a city in eastern Ethiopia with a population of around 289,000 people with six urban and three peri-urban woredas (third level administrative divisions, equivalent to districts) [19]. As Pull-Push project focus was on urban food systems we selected the six urban districts, comprising 19 kebeles (fourth level administrative divisions in Ethiopia, e.g. ward or neighbourhood) with 30,310 households. A detailed description of Harar in the context of the present study is available in previous studies from the same project [4], [6].

### 2.3 Study design

**Fig. 1:**
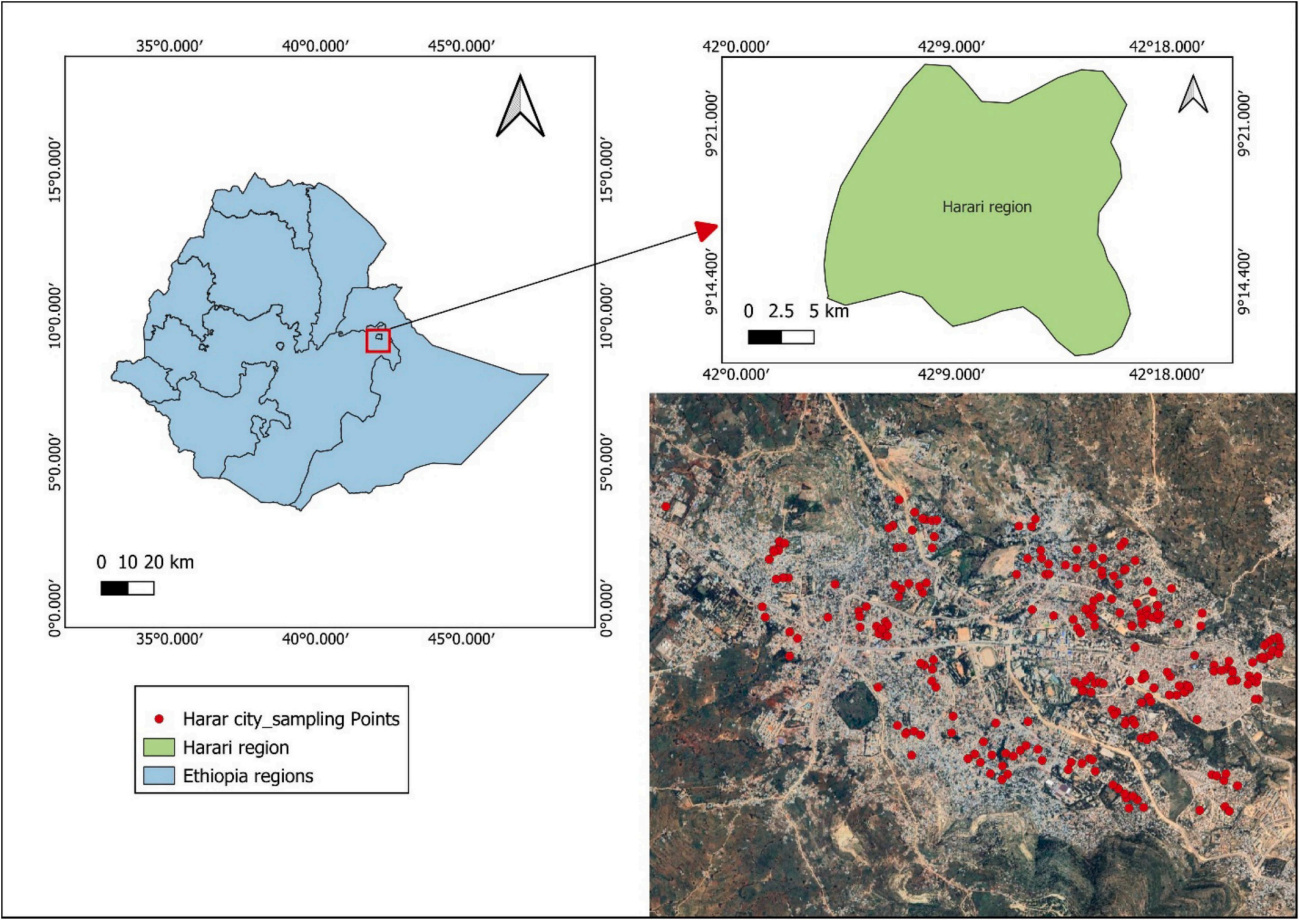
Geographic distribution of study households in Harar city, Ethiopia.

We conducted a cross-sectional survey with random sampling from the sampling frame of all the households in the six urban woredas (districts) (Fig. 1). The list of households was provided by the public health extension office under Harar regional health bureau. Non-response was addressed through systematic replacement with the next available neighbour from the sampling frame. (See Table 1.)

**Table 1:** Socio-economic and demographic characteristics of households in Harar, Ethiopia.

Variable	Category	% (*n* = 252)	95% CI
Household Head	Male-headed	63%	57.03–68.96
	Female-headed	37%	31.04–42.96
Education Level of the respondent	No formal education	14%	9.72–18.28
	Elementary school	29%	23.4–34.6
	Secondary education	27%	21.52–32.48
	Post-secondary (TVET/Uni)	19%	14.16–23.84
Household Size	One to four people	71%	65.42–76.92
	Five or more people	29%	23.08–34.57
Children <5 years in household	At least one	45%	38.86–51.14
Elderly (≥65 years) in household	At least one	19%	14.16–23.84
Primary household income source	Trade	30%	24.34–35.66
	Government employment	35%	29.11–40.89
	Informal work	30%	24.34–35.66
Housing type	Own home	45%	38.86–51.14
	Private rental	18%	13.26–22.74
	Municipal rental	37%	31.04–42.96
Self-reported socio-economic status range 1–10	Low (1–2)	32%	26.05–37.88
	Medium (3–5)	62%	56.00–68.31
	High (6–8)	6%	3.34–9.63
Separate kitchen	Yes	75%	69.65–80.35
Water source	Municipal piped water	94%	91.07–96.93
	Well	4%	2.20–7.68
	River	1%	0.25–3.44
Asset ownership	House	44%	38.2–50.8
	Refrigerator	65%	59.11–70.89
	Deep freezer	1%	0–2.23
	Car	2%	0.27–3.73
	Motorized three-wheeler (*bajaj*)	6%	3.07–8.93
Source of food safety information	Television	76%	70.73–81.27
	Friends/Relatives	48%	41.83–54.17
	Social media	15%	10.59–19.41
	Other	10%	6.3–13.7
	None	5%	2.31 - 7.69

### 2.4 Study population

The study exclusively recruited women as respondents because preliminary formative research and cultural norms in the study area indicated that women are almost universally responsible for household food preparation and cooking. This gender-specific recruitment strategy ensured that we captured the practices of the household members actually performing food handling tasks.

### 2.5 Sample size

The sample size was determined using the standard formula for estimating a proportion in a large population, assuming that 20% of household food handlers would have good KAP, with a 95% confidence level and a 5% margin of error. The initial calculation yielded *n* = 246. To account for an anticipated 3% of responses missing or being unusable, the final sample size was increased to 252 households.

### 2.6 Survey instruments

A structured questionnaire was developed based on a qualitative investigation of chicken and vegetable value chains in the present study area. The questionnaire contained questions on socio-economic and demographic characteristics, hygiene infrastructure within households, chicken and tomato purchasing habits, practices for preparing food within the household and consumption, and other pertinent information. The questionnaire was originally developed in English to be delivered in Kobo Toolbox and translated into Amharic (the official language of Ethiopia) and Afaan Oromoo (the local language of Harar), and back translated to English to ensure accuracy. Questionnaires were administered in the respondent's preferred language by trained enumerators fluent in both languages. Before conducting the study, the questionnaire was pretested with food handlers from eight households, which were not included in the final survey, and minor adjustments were made. The informed consent form and complete questionnaire are provided in Supplementary Material 1, Supplementary Material 2 respectively.

### 2.7 Data collection

Data were collected during the main rainy season (*Kiremt*; June–July) in 2023. Seasonal variations in food availability, prices, and handling practices may exist, however, both chicken and tomatoes are available year-round in urban markets, and core hygiene practices are expected to be relatively stable across seasons. The questionnaire was administered to respondents in Amharic or Afaan Oromoo in face-to-face interviews and the data collected using KoboToolbox software. The data for this study was collected by four trained enumerators. Each enumerator was provided with a list of selected households to visit, with a reserve list available to replace those who were not available or did not consent to the survey or were unreachable. Prior to conducting the survey, the enumerators clarified the objective of the work and obtained a written informed consent from each participating household.

### 2.8 Data management and analysis

Data were cleaned, coded, and analysed using Stata version 17 [20]. Descriptive statistics were computed for all variables. Household food hygiene practices were independently classified by two investigators with expertise in veterinary public health and food safety and extensive experience in Ethiopian food value chains. Each investigator independently categorized practices as “good,” “risky,” or “neutral” based on microbiological principles (including cross-contamination pathways, time–temperature control, environmental contamination risk) and alignment with international guidance, including the WHO Five Keys to Safer Food framework [21]. Classifications were compared and discrepancies resolved through structured discussion and justification based on contamination-risk assessment in the local context. Only one indicator required deliberation. The final categorization was reviewed by co-authors who concurred with the categorization.

The HGFHP Index was conceptualized as a formative composite measure in which each indicator represents a distinct behavioural or environmental dimension contributing to food safety risk. Indicators were therefore not assumed to covary uniformly or represent a single latent construct. Indicator selection was guided by microbiological risk pathways identified in the literature, including routes of cross-contamination, hygiene practices, and environmental exposure, as well as expert judgment. A detailed list of indicators, scoring criteria, and aggregation procedures is provided in Supplementary Material 3. Each indicator was assigned a categorical score (good = 1, risky = −1, or neutral = 0), and equal weighting was applied in constructing the composite index. The index was calculated by aggregating individual indicators to provide an overall measure of household food safety practices. 233. Composite indices aggregating multiple food safety behaviours have been increasingly used in LMIC settings to provide holistic assessments of food safety practices [22]. These indices offer advantages when used together with single-item measures by capturing the multidimensional nature of food safety behaviours and allowing for more comprehensive risk stratification. The analytical framework was informed by the Knowledge, Attitudes, and Practices (KAP) and the COM-B model (Capability, Opportunity, Motivation-Behaviour). Predictor selection was guided by literature review, expert judgment, and a directed acyclic graph (DAG) to minimize over-adjustment bias. Exploratory analysis indicated substantial clustering at the kebele level and minimal clustering at the woreda level, but nesting was retained for structural consistency; multilevel linear regression with random intercepts for kebeles was therefore used to account for the hierarchical structure of the data. Prior to fitting the linear regression models, assumptions were assessed, including normality of the outcome and residuals, homoscedasticity, influence of outliers, and multicollinearity among predictors. All findings are interpreted as associative and hypothesis-generating, consistent with the cross-sectional design. Complete methodological details are provided in Supplementary Material 3.

### 2.9 Conceptual framework

**Fig. 2:**
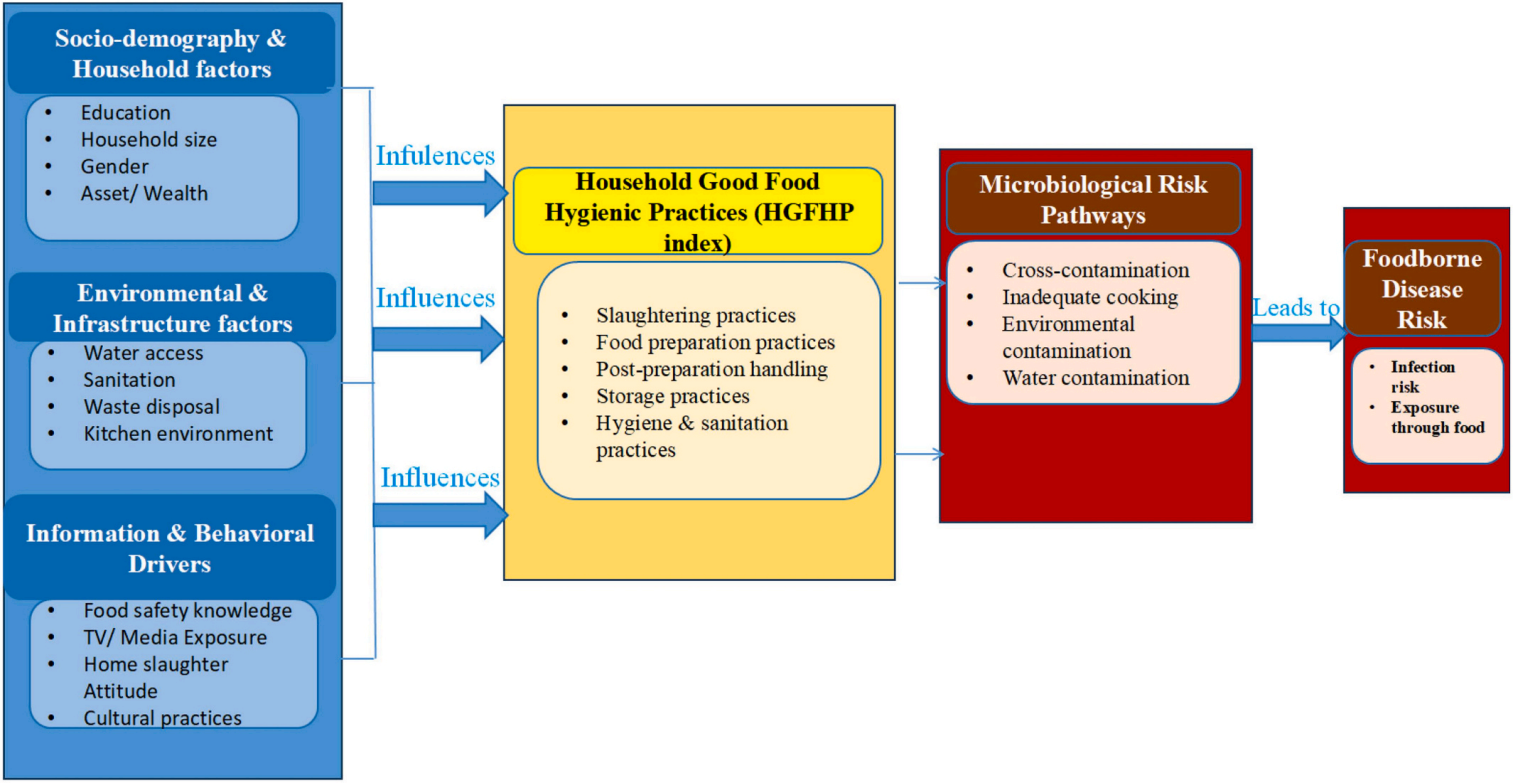
Conceptual framework of household food hygiene practice and foodborne disease risk pathway.

The framework illustrates how practices across pre-processing, processing, and post-processing stages contribute to the introduction, transfer, and amplification of foodborne pathogens. Key pathways include cross-contamination between raw poultry and ready-to-eat foods, inadequate utensil and surface hygiene, unsafe water use, and improper storage conditions. Each component represents an independent contributor to contamination risk, consistent with the formative structure of the HGFHP Index (see the diagram in Fig. 2).

### 2.10 Ethical considerations

Ethics approval was obtained from the Institutional Research Ethics Committee of the International Livestock Research Institute, Ref: ILRI-IREC2023–20. Relevant local and national permissions were sought and obtained.

## 3 Results and discussion

### 3.1 Results

#### 3.1.1 Demographic and socio-economic characteristics of participants

A total of 252 households participated in the study, with an 85% response rate. As per the study design, non-respondents were replaced by willing substitutes households from the sampling frame. Most households were male headed, but all respondents were women. Mean respondent age was forty-one years, with female-headed households having older respondents than male-headed households.

Households had an average of nearly five members, with about half having children under five and one-fifth including elderly members, both groups most vulnerable to foodborne disease. Educational attainment varied: approximately one-fifth of household heads completed post-secondary education, while female-headed households tended to have slightly lower educational levels.

Regarding economic status, nearly two-thirds lacked formal employment, while about 30% were engaged in trade. Homeownership was reported by less than half, while others rented from private landlords or municipal housing. Self-reported socioeconomic status was generally low-to-moderate levels, with a small proportion at the lowest level and none in the highest categories.

Most households had separate kitchens, and nearly all had access municipal piped water, although the majority experienced frequent interruptions. During these disruptions, most relied on alternative safe water sources, while only a small use river water. Television ownership was universal, and nearly two-thirds of households owned refrigerators. However, less than half owned houses, with small proportions owning farmland, three-wheelers, cars, and deep freezers. Asset ownership correlated moderately with self-reported socioeconomic status.

Most participants reported having access to information on safe food handling and preparation. Television was the most common source, reported by 76% of households, followed by social networks such as friends and relatives (48%).

#### 3.1.2 Household chicken and tomato purchasing and consumption behaviour

##### 3.1.2.1 Chicken purchasing practices

Regarding chicken purchase practices, the vast majority of households reported purchasing live chickens from markets for home slaughter, as shown in Table 2. Slaughtering backyard-raised chickens was less common, and the purchase of pre-slaughtered or frozen chicken was rarely reported. When frozen chicken was purchased, come from both Central Ethiopia and imported source.

**Table 2:** Chicken and tomato purchase, preparation and consumption practices of the surveyed households.

**Practice**	**(%)**
Chicken Source	Purchase live birds from market and slaughter them at home (92%), Slaughter own chicken in backyard at least sometimes (21%), Buy frozen at least sometimes (6%), Buy and get slaughtered at market sometimes (2%)
Chicken Preparation	*Doro wat* (100%) Roasted (12%); with rice (8%); salad (4%); soup (2%)
Chicken Consumption	Infrequent (1.7 times/month); SD 5.2, range 0–30
Tomato Preparation and consumption	92% ate tomatoes daily 85% sauce; 26% salad and 18% slightly warmed

##### 3.1.2.2 Chicken and tomato preparation preferences and consumption frequency

All households prepared chicken as *Doro wat* (traditional Ethiopian stew), with alternative less common preparations being roasting and chicken with rice. Other preparation methods were rare. Chicken consumption was infrequent outside of holiday periods (Table 2). Households with higher chicken consumption frequency had significantly higher self-perceived socioeconomic status (mean = 4.0 vs. 3.2, *p* = 0.0004).

In contrast, tomato consumption was nearly universal, with 92% reporting daily consumption. Tomatoes were primarily prepared and consumed cooked in sauces, with 87% always or usually consuming sauce preparations. Raw tomato consumption was less frequent (26% always or usually), while *leb-leb* (slightly warmed tomato) preparation was least common (19% always or usually) (Table 2). While tomatoes were consumed an average of 6.7 days per week (SD 1.3, range 0–7), raw tomatoes in salads were consumed an average of 2.4 times per week.

#### 3.1.3 Household food preparation and hygiene practices

Household food preparation practices were assessed in relation to risk-mitigating or risky behaviours, as categorized by study experts based on established food safety principles. Chicken hygiene practices were grouped into three process-related practices (slaughter, carcass preparation, and post-preparation) and three equipment- and infrastructure-related practices (cold storage, water source, utensils, and surfaces). Tomato hygiene practices were categorized into three process-related practices (storage, preparation, and post-preparation) and one equipment-related practice (utensils). These classifications contributed to the development of the HGFHP Index.

#### 3.1.4 Chicken hygiene practices

##### 3.1.4.1 Slaughtering practices

As shown in Table 3, the majority of households purchased live chickens from markets and slaughtered them at home rather than at the point of purchase. Most households kept live birds for extended periods before slaughter, which may increase the risk of contamination. Despite this practice, awareness of the risk associated with home slaughter was low; most participants perceived no problems and intended to continue the practice due to cultural preferences. Slaughter locations varied between indoor and outdoor settings, and waste disposal practices were inconsistent. However, nearly all respondents reported avoiding the slaughter of visibly sick birds.

**Table 3:** Hygiene practices for chicken and tomato performed by different households (percentage of households performing a practice shown in brackets).

**Category**	**Risk-Mitigating Practice**	**Risky Practice**
Chicken		
Slaughter location	Home slaughter (92%)	Market slaughter (1.98%)
Live chicken household storage	Slaughtered day of purchase (14%)	Kept for >1 day (86%)
Slaughtering location	Outdoors (42%)	Indoors (58%)
Carcass cleaning	Washed with lemon (96%)	Not always washed (4%)
Marination time	>30 min (41%)	<30 min (59%)
Cutting board use	Separate board for meat and vegetables (26%)	Same board for meat and vegetables (74%)
Handwashing after prep	Always (67%)	Not always (33%)
Cold storage of chicken carcase	Bottom shelf (15%)	Any shelf (85%)
Storage of leftovers at room temperature	<2 h (42%)	>2 h (58%)

Tomato		
Storage temperature	Refrigerated (44%)	Room temp (56%)
Washing tomatoes	With soap (45%)	With water only (55%)
Storage of tomato sauces at room temperature	<2 h (19%)	>2 h (81%)

##### 3.1.4.2 Carcass preparation practices

Carcass preparation practices varied considerably among households (Table 3). Among respondents, 96% reported washing chicken carcasses, using lemon juice. This practice reflects traditional food preparation methods believed to improve taste and cleanliness, and to reduce odours. However, marination times were generally too short to achieve meaningful food safety benefits. Traditional practices such as using pea powder to scrub the chicken skin were also common, although their effectiveness in reducing microbial contamination remains unclear. Cross-contamination risks were evident through the use of the same cutting board for both raw chicken and vegetable preparation. Hand hygiene practices were inconsistent, with only two-thirds of respondents washing their hands immediately after handling raw chicken.

##### 3.1.4.3 Post-preparation practices

Chicken was most often consumed immediately after cooking. However, 38% of households left food at room temperature for more than two hours before serving, increasing the risk of bacterial growth. Leftovers were commonly stored, but 58% had kept them at room temperature for more than two hours before refrigeration or reheating.

#### 3.1.5 Equipment and infrastructure-related practices

##### 3.1.5.1 Cold storage practices

While most households owned refrigerators, proper storage practices were uncommon (Table 3). The majority stored raw chicken on upper or middle shelves instead of the recommended bottom shelf, increasing cross-contamination risks. Additionally, over one-fifth kept raw chicken refrigerated for extended periods, potentially promoting bacterial growth.

##### 3.1.5.2 Water source and cleaning practices

Although 94% of households had access to municipal piped water, 38% relied on stored water in open containers during supply interruptions, which poses a risk of contamination. Kitchen surfaces were cleaned after food preparation in 65% of households, a risk-mitigating practice, while 35% did not clean surfaces consistently, a risky practice. Nearly all respondents (98%) cleaned utensils with soap, but only 30% used hot water.

#### 3.1.6 Tomato hygiene practices

##### 3.1.6.1 Storage practices

Most tomatoes were stored at room temperature, while less than half were refrigerated (Table 3). The first tomato used for salads was kept for an average of 1.04 days, while the last tomato used for sauce was stored for up to 9.6 days, indicating potential risks if storage conditions are inadequate.

##### 3.1.6.2 Preparation practices

While 94% of respondents reported washing tomatoes before consumption, only 45% used soap or any form of disinfectant. Additionally, 66% did not separate damaged tomatoes from intact ones, increasing the risk of spoilage and microbial contamination. About 74% used the same cutting boards for vegetables and raw meat, further increasing cross-contamination risks.

##### 3.1.6.3 Post-preparation practices

Among the respondents, 26% reported consumed raw tomatoes, often in salads, while, 18% consumed slightly warmed tomato. When combined with inadequate separation of raw chicken meat and vegetable preparation, these practices creates a significant risk of microbial cross-contamination. In addition, the majority of respondent (81%) stored tomato-based sauces at room temperature for more than two hours before consumption, which could promote bacterial growth.

##### 3.1.6.4 Utensil cleaning practices

Although 98% of households cleaned utensils with soap, only 30% used hot water for disinfection, and 35% did not consistently clean food preparation surfaces after handling raw ingredients.

#### 3.1.7 Household good food hygienic practice index

The Household Good Food Hygienic Practices (HGFHP) Index was initially developed using 44 indicators capturing household food hygiene behaviours and infrastructure related to chicken and tomato handling (Supplement Material 5). These indicators spanned multiple domains, including slaughtering, preparation, post preparation handling, storage, and household infrastructure. The indicators were selected to reflect epidemiologically relevant practices along contamination pathways rather than a single underlying construct.

**Fig. 3:**
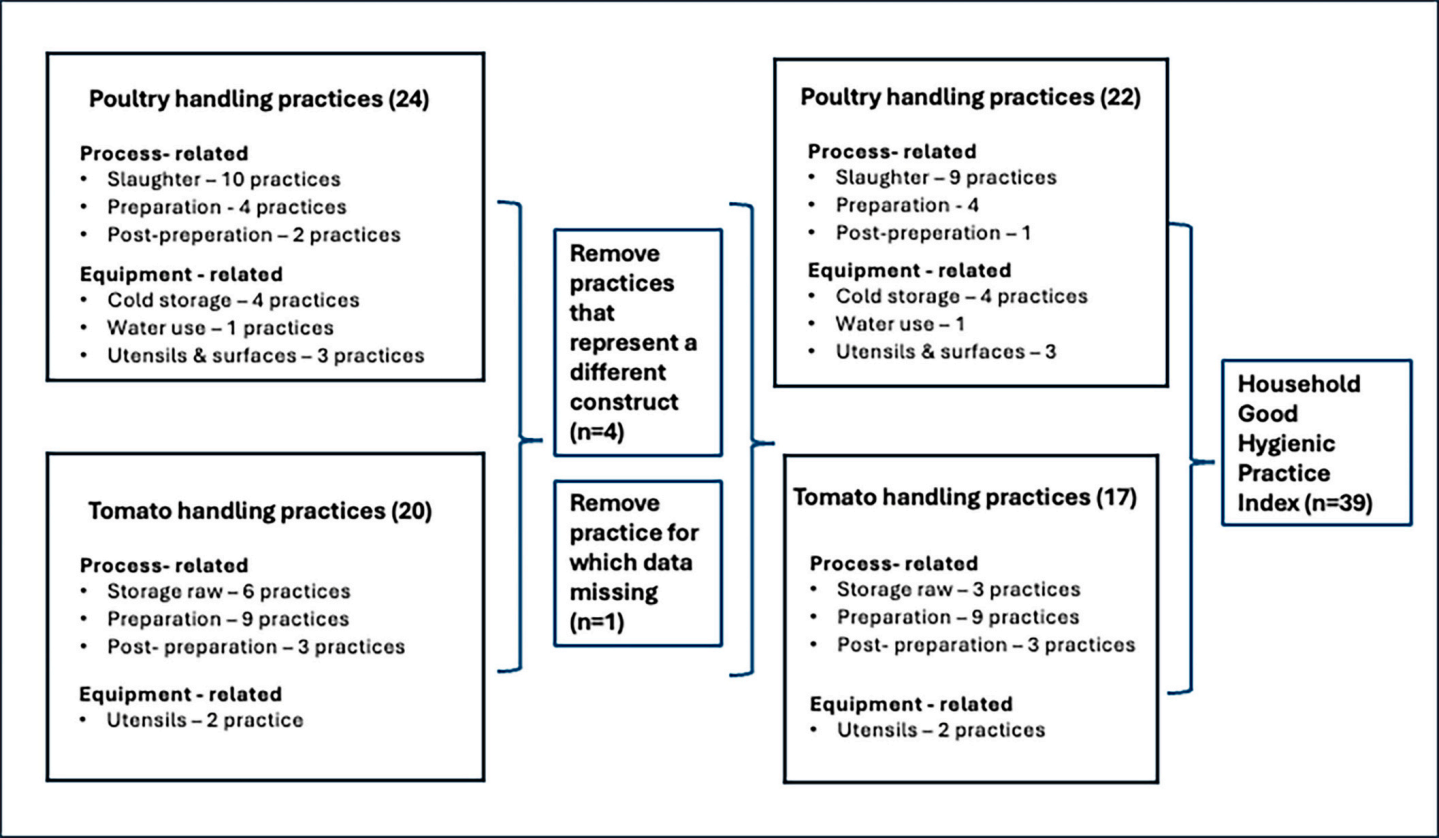
Number of variables for chicken and tomato household food safety and hygiene practice index.

During index development, Cronbach's alpha was calculated as a descriptive diagnostic statistic to examine the behaviour of the indicator set. The Initial assessment of Cronbach's alpha suggested that four variables (days the chicken was kept at home before slaughter, place of tomato storage, and time tomatoes were kept at home before use for salad and for sauce) showed weaker alignment with the remaining indicators. These variables were excluded to improve conceptual clarity and consistency of the index. Following refinement, the final HGFHP Index consisted of 39 variables, covering slaughtering, preparation, post-preparation, storage, and household infrastructure (Fig. 3). The Cronbach's alpha value for the final set was 0.8175 and is reported here for descriptive purposes only, without implying internal consistency, reliability, or unidimensionality, in line with the formative nature of the index. The sub-indices for chicken and tomato hygiene were positively correlated (*r* = 0.62, *p* < 0.001). The distribution of the HGFHP scores approximated normality.

#### 3.1.8 Multi-level modelling of HGFHP scores

A multiple linear mixed-effects regression model was applied to examine the socio-economic variables associated with household hygienic practices. The HGFHP score was significantly and positively associated with four mains variables (Table 4): Living in an area with good infrastructure, receiving food safety information from television, attitude toward no problem with home slaughter, and having no education.

**Table 4:** Mixed-effects regression results showing factors associated with HGFHP scores, reflecting food safety handling practices.

**Variable**	**Coefficient (β)**	**Std. errs.**	**P**	**95% Confidence Interval**
Woreda infrastructure rated “good”	5.22	2.29	0.023	0.73–9.71
Male-headed household	−0.32	0.90	0.720	−2.09 - 1.44
Age of respondent	−0.02	0.03	0.491	−0.09 - 0.04
Number of people in household	0.47	0.22	0.035	0.03–0.91
No education	2.71	1.13	0.016	0.50–4.92
Asset owned (at least one)	−1.26	0.91	0.169	−3.04 - 0.53
No problem with home slaughtering	4.23	0.91	0.000	2.44–6.02
Gets food safety information from TV	4.53	0.92	0.000	2.73–6.33
Constant	12.37	2.84	0.000	6.80–17.94

Random intercept for kebele nested within woreda (kebele_in_woreda) *N = 252, Groups = 18.*

Observation per group: Min = 1, Avg = 14, Max = 25.

Random Effects = Intercept (kebele_in_woreda): Var = 0.112,

Model Statistics = Log Likelihood = −818.55584, suggesting that the mixed-effects specification effectively captured the hierarchical structure of the data. Wald χ^2^ (8) = 78.93, The model demonstrated a good overall fit, Prob > χ^2^ = 0.000.

In addition, a mixed-effects maximum likelihood regression model was fitted to examine the association between good food safety handling practices and various household and individual-level factors. These, included woreda infrastructure rating, gender of household head, age, household size, no educational attainment, asset ownership at least one, no problem with home slaughtering risks, and access to food safety information via television, with kebele (ward) nested within woreda (district) specified as a random effect.

### 3.2 Discussion

This study provides an assessment of household food hygiene practices for chicken and tomato handling in an urban Ethiopian. FBD is a major One Health issue, and the findings emphasise the importance of household environments, and human behaviour in determining zoonotic and non-zoonotic foodborne disease risks, including through cross-contamination of pathogens between animal sourced foods and vegetables.

Overall, substantial variation was observed across households, with that food hygiene practices influenced by a combination of behavioural, socio-economic, and informational factors rather than a single underlying factors.

The study found that 92% of households purchased live chickens from markets and slaughtered them at home, while 19% also slaughtered backyard poultry. This strong preference for live poultry over pre-slaughtered chicken aligns with studies in East Africa showing that freshness, quality control, and cultural significance drive consumer preference for live birds [23]. Similar trends have been reported in Nigeria and Kenya, where consumers prefer freshly slaughtered meat due to trust deficits in formal supply chains and concerns over hygiene in pre-packaged poultry [24], [25]. However, from a microbiological perspective, home slaughter introduces multiple contamination risk, including exposure of carcasses to environmental pathogens, inadequate separation of clean and dirty zones, and limited access to sanitation infrastructure. Similar household-level risks have been documented in the Eastern Mediterranean region, where studies in Lebanon identified cross-contamination during household meat preparation as a critical driver of foodborne disease risk [16]. These pattern across regions highlight the global relevance of household-level food hygiene practices as critical control points in the prevention of foodborne disease. Consumer behaviour, including preferences for perceived freshness and trust in informal markets, has been shown to influence food safety practices and risk exposure. Evidence shows that perceptions of quality, hygiene, and trust shape purchasing and handling decisions, ultimately affecting food safety outcomes downstream [15], [24], [26]. The continued reliance on home slaughter, despite associated potential food safety risks, suggests deeply rooted cultural practices and a lack of accessible, trusted alternatives [27].

The relatively low frequency of chicken consumption (1.7 times per month outside holidays) reflects economic constraints, as poultry remains an expensive protein source for many low-income households. This supports findings from Ethiopia and other LMICs where chicken and other ASFs are often consumed primarily during religious or festive occasions [10].

In contrast, tomato consumption was almost universal (92% daily intake), reinforcing its central role in Ethiopian cuisine, particularly as a base for traditional sauces and stews. Cross-contamination risks were evident in kitchen hygiene practices. Only 26% of households used separate cutting boards for raw chicken and vegetables, and 67% reported consistent handwashing after handling raw poultry, both considered as risk-mitigating practices. However, 74% of households used the same cutting boards for raw chicken and other foods, and 35% did not consistently clean food preparation surfaces, increasing cross-contamination risks. These findings align with established evidence that inadequate separation of raw and cooked foods is a major source of foodborne illness globally [21], [28].

The high prevalence of raw tomato consumption (71%) further amplifies this risk, as fresh produce can act as a vehicle for pathogen transmission when contaminated during preparation with no pathogen kill-step before consumption. Similar observations have been reported in LMICs and the Eastern Mediterranean region, where poor kitchen hygiene practices contribute to contamination of fresh vegetables consumed raw [16], [29].

From a food safety risk perspective, food hygiene practices varied considerably, with both risk-mitigating and risky behaviours identified. Marketplace slaughter (2%) and indoor slaughter (58%) were classified as risky practices, whereas slaughtering chickens outdoors (92%), and washing carcasses with lemon juice (96%) were categorized as risk-mitigating behaviours. The latter is a common traditional practice in Ethiopian cuisine believed to both improve flavour and cleanliness. While acidic marinades can reduce surface bacterial loads to some extent [30], this practice does not eliminate pathogens and may increase cross-contamination risk if wash water splashes onto kitchen surfaces or ready-to-eat foods. Importantly, such washing practices do not substitute adequate cooking, which remains the critical control point for eliminating foodborne pathogens. Food safety education should acknowledge these traditional practices while emphasizing proper cooking and prevention of cross-contamination during preparation.

Cold storage practices were suboptimal. Only 15% of households stored raw chicken on the bottom refrigerator shelf, and 22% kept it for more than one day, both categorized as risky behaviours. This is particularly concerning given that improper refrigeration is a leading risk factor for bacterial growth and foodborne disease transmission [31]. Similar findings have been reported in LMICs where refrigeration infrastructure is limited or inconsistently used [32].

Water access also played a critical role in hygiene practices. While 94% of households had municipal piped water, 38% relied on stored water in open containers during supply interruptions, a risky practice that increases microbial contamination risks. This is consistent with studies showing that water storage in open containers is a key risk factor for foodborne pathogen transmission in Ethiopia [33].

The study also developed the HGFHP Index as a formative composite measure to capture multiple dimensions of household food hygiene behaviour and infrastructure. The positive correlation between hygiene scores for chicken and tomatoes (*r* = 0.62, *p* < 0.001) suggests that households that practice good hygiene in one food category tend to do so across food types. This support evidence that food safety behaviours are often generalized rather than food-specific [22]. The index therefore provides a useful tool for assessing overall household food safety performance, while recognizing the multidimensional nature of hygiene practices. These findings underscore the importance of integrated household-level hygiene interventions that address both animal-source foods and raw vegetables rather than focusing on single commodities.

Regression analysis identified four key variables associated with higher household hygiene scores: living in an area with good infrastructure (β = 5.22, *p* = 0.023), receiving food safety information from television (β = 4.53, *p* < 0.0001); perceiving no problem with home slaughter (β = 4.23, p < 0.0001); and having no educational background (β = 2.71, *p* = 0.016). Living in areas with better infrastructure was strongly associated with improved household hygienic practices (β = 5.22). This finding aligns with evidence from previous studies indicating that basic infrastructure, particularly access to clean water, sanitation, and waste disposal systems, is essential for enabling and sustaining good food hygiene behaviours [15]. In low-resource settings, inadequate infrastructure has consistently been linked to increased risk of foodborne illness due to limited options for safe washing, storage, and disposal. Even if individuals have knowledge and motivation to follow good hygienic practices, the absence of an enabling environment, such as functional water supply or proper drainage, can constrain behaviour.

Similarly, the effect of television as an information source aligns with previous studies showing that mass media campaigns significantly influence hygiene-related behaviours in LMICs [34]. Television ownership and neighbourhood infrastructure also correlates with broader socio-economic factors such as wealth, and education. Evaluation of a multi-media food safety awareness campaign conducted in the Pull–Push project in Dire Dawa and Harar, Ethiopia found that, recall of television-based food safety messages was associated with safer food purchasing and preparation practices, as well as improved knowledge, intentions, and self-efficacy among women consumers [35], while similar associations with consumer knowledge and intentions were observed in Ouagadougou, Burkina Faso [36]. These results highlight the potential of television based, culturally tailored mass media campaigns as scalable and cost-effective components of integrated food safety interventions in urban informal food systems.

Participants who did not perceive home slaughter as problematic demonstrated significantly higher household good food hygienic practice scores (β = 4.30). Although this may seem counterintuitive, given the general assumption that formal slaughtering facilities provide safer meat, it may reflect contextual realities in LMICs [37]. In many LMICs, including Ethiopia, formal slaughterhouses and market are often inadequately regulated and poorly equipped, increasing the risk of microbial contamination during processing, transportation, and sale [37], [38]. As a result, some households with stronger food safety knowledge and practices may prefer home slaughter, perceiving it as a safer and more controllable option. Furthermore, home slaughter is deeply embedded in local cultural and religious traditions, often tied to festivals, communal sharing, and long-standing practices. These traditions may not only normalize but also reinforce hygienic behaviours at the household level. Previous studies have noted that traditional practices, when combined with local knowledge and responsibility, can contribute to effective hygiene management in informal settings [15]. Therefore, rather than indicating a lack of concern for food safety, the acceptance of home slaughter in this context may reflect a pragmatic and culturally informed response to perceived risks in the formal sector.

Interestingly, the analysis revealed that participants with no formal educational background had significantly higher HGFHP scores compared to those with some education (β = 2.71). This does not suggest a protective effect of limited education, but may reflect the role of experiential learning, traditional food handling knowledge, and routine domestic engagement with food preparation among women with limited formal schooling. Similar findings have been reported in other LMIC settings, where formal education does not necessarily improve food safety practice without targeted hygiene training [39], [40]. This contrasts with studies showing a positive association between education and food hygiene practices (e.g., [41], [42]. For example, Al Banna et al., [41] found that formal education improves food safety awareness and compliance with hygienic practices among food handlers. The positive association between lack of formal education and better hygiene scores is counterintuitive but may be explained by several mechanisms, including experiential and intergenerational knowledge transmission, strong community norms and social learning, and a cohort effect whereby older women accumulate extensive practical food handling experience. In addition, more educated individuals may face time constraints that limit careful food preparation, and formal education rarely includes food safety training. Together, these findings suggest that experiential learning may be more influential than general education for developing practical food safety skills and that interventions should build on, rather than replace, existing traditional knowledge.

To the best of our knowledge, this study represents the first examination of household food safety practices related to chicken and tomatoes in Ethiopia. The study provides valuable insights into food hygiene behaviours in urban households yet certain methodological considerations should be acknowledged.

Recall bias is likely minimal, as participants reported routine behaviours. However, response bias is a concern, as participants may provide socially desirable answers rather than fully accurate responses such as claiming to wash hands after using to the toilet. The potential for interviewer effects, where respondents adjust their answers based on perceived expectations, should also be acknowledged. An additional, as yet unpublished study using direct observation among a subset of participants suggests that observed practice may provide more accurate data than self-reported.

The cross-sectional design limits causal inference. For example, although television-based food safety education was associated with higher hygiene scores, the study cannot establish whether exposure to food safety messages directly improved hygiene behaviours or whether households that already prioritized food safety were more likely to watch such programs. Future experimental or longitudinal studies would help establish these causal relationships.

## 4 Conclusion

This study contributes to the limited evidence on household food hygiene practices in urban Ethiopia, focusing on the handling of chicken and tomatoes. The findings highlight the central role of women in food preparation and hygiene management, underscoring the importance of designing gender-sensitive food safety interventions. The continued reliance on informal markets for animal-source foods supports nutritional resilience but also presents challenges for pathogen control. The strong correlation between hygienic practices for chicken and tomatoes suggests that food safety interventions should target cross-cutting behavioural practices rather than specific food items. Moreover, access to food safety information, particularly through television and social networks, was associated with improved hygiene, emphasizing the critical role of public health education and communication strategies. Mass media-based food safety education campaigns, particularly through television, should be prioritized as a scalable intervention strategy reaching diverse populations regardless of education or wealth status. The observed differences in hygienic practices according to educational and attitudes toward home slaughter further indicate the need for culturally tailored and context-specific interventions. From a One Health perspective, the findings demonstrate how interactions among animal-source foods, household environments, and human behaviour shape foodborne disease risks at household level. Integrated interventions that simultaneously address food handling practices, environmental hygiene, and food safety awareness are therefore needed to reduce pathogen transmission and improve public health outcomes. Finally, by identifying key variables associated with hygienic behaviour in an index, this study provides actionable, evidence-based insights for policymakers, public health professionals, and food safety advocates. The Index also has potential for broader application in LMICs to support standardized monitoring and food safety intervention design.

The following are the supplementary data related to this article.

Supplementary Material 1 Informed consent form (Afan Oromoo and Amharic versions)

Supplementary Material 2 Complete questionnaire (English versions)

Supplementary Material 3 Complete details of statical methods used in the study data analysis.

Supplementary Material 4 Lists of variables for chicken and tomato household food safety and construction of hygiene practice index

## Declaration of generative AI and AI-assisted technologies

During the preparation of this work the author(s) used Paperpal, an AI-based academic language editing program to improve the clarity of the written text. The author(s) have reviewed and edited the content further and take(s) full responsibility for the content of the manuscript.

## CRediT authorship contribution statement

**Meseret Bekele:** Writing – review & editing, Writing – original draft, Visualization, Software, Formal analysis, Data curation. **Kebede Amenu:** Writing – review & editing, Validation, Supervision, Methodology, Conceptualization. **Biruk Alemu Gemeda:** Writing – review & editing, Validation, Methodology, Investigation. **Johanna F. Lindahl:** Writing – review & editing, Validation, Supervision. **Mohammed Ahmed:** Writing – review & editing, Investigation. **Sisay Girma:** Writing – review & editing, Validation, Supervision, Methodology. **Theodore J.D. Knight-Jones:** Writing – review & editing, Validation, Supervision, Project administration, Methodology, Conceptualization. **Delia Grace:** Writing – review & editing, Validation, Supervision, Methodology, Funding acquisition, Formal analysis, Data curation, Conceptualization.

## Funding

This study was funded by the 10.13039/100000865Bill and Melinda Gates Foundation, UK Government Foreign, Commonwealth and Development Office (FCDO)-UK Aid from the United Kingdom government (INV-008430-OPP1195588), One Health Centre in Africa (OHRECA) and the CGIAR Research Program on Agriculture for Nutrition and Health. The CGIAR Sustainable Animal and Aquatic Foods (SAAF) Science Program also supported this study during its preparation/writing. Any opinions, findings, conclusions, or recommendations expressed here are those of the authors.

## Declaration of competing interest

The authors declare that they have no known competing financial interests or personal relationships that could have appeared to influence the work reported in this paper.

## Data Availability

The data supporting this study are accessible upon request from the authors.

## References

[1] John Mususa Ulimwengu J.M., Kwofie E.M., Collins J. (2023).

[2] Shludg Steven Jaffee, Ec (2019).

[3] van Wagenberg C.P.A., Havelaar A.H. (2023). Economic costs related to foodborne disease in Burkina Faso and Ethiopia in 2017. Front. Sustain. Food Syst..

[4] Amenu K., Bedasa M., Wamile M., Worku H., Kasim K., Taha M. (2021).

[5] Afonso A., Blanco-Arana M.C. (2024). Does financial inclusion enhance per capita income in the least developed countries?. Int. Econ..

[6] Gemeda B.A., Amenu K., Girma S., Grace D., Srinivasan R., Roothaert R. (2023). Knowledge, attitude and practice of tomato retailers towards hygiene and food safety in Harar and Dire Dawa, Ethiopia. Food Control.

[7] Grace D. (2023).

[8] FAO, UNEP, WHO, WOAH (2022).

[9] Gazu L., Alonso S., Mutua F., Roesel K., Lindahl J.F., Amenu K. (2023). Foodborne disease hazards and burden in Ethiopia: a systematic literature review, 1990–2019. Front. Sustain. Food Syst..

[10] Milkias M. (2016). Chicken meat production, consumption and constraints in Ethiopia. Food Sci. Quality Manag..

[11] Dugassa G. (2022). Current status of poultry meat production, processing and Marketing in Ethiopia: a review. J. Anim. Husbandr. Dairy Sci..

[12] Observatory of Economic Complexity (OEC) (2024). Poultry in Ethiopia Trade | The Observatory of Economic Complexity. https://oec.world/en/profile/bilateral-product/poultry/reporter/eth.

[13] TrendEconomy. Ethiopia | imports | meat and edible offal, of the poultry | 2023. Https://TrendeconomyCom/ 2024. https://trendeconomy.com/data/import_h2/Ethiopia/0207 (accessed June 28, 2026).

[14] Redmond E., Curnin M.A., Day M.C., Mbe J.M., Eves A., Raats M. (2018).

[15] Roesel K., Grace D. (2014).

[16] Karam J., Serhan M., Haddad C., Sacre H., Jomaa L., Salameh P. (2024). Exploring food safety knowledge and practices in Lebanon. East Mediterr. Health J..

[17] International Livestock Research Institute (ILRI) (2019).

[18] Haile D., Tesfaye B., Assefa F. (2022). Overview of agrochemicals application practices on tomato farm by smallholders at Koka, Meki and Ziway, Ethiopia. Turkish J. Agricult. - Food Sci. Technol..

[19] Ethiopian Statistics Services Population Projection 2024 | Ethiopian Statistics Services 2024. https://ess.gov.et/download/projected_population-2024/.

[20] StataCrop (2021). STATA INDEX RELEASE 17. Published by Stata Press, 4905 Lakeway Drive, College Station, Texas 77845 Typeset in TEX. https://www.stata.com/manuals17/i.pdf.

[21] WHO (2006). https://www.who.int/publications/i/item/9789241594639.

[22] Medeiros C.O., Cavalli S.B., Salay E., Proença R.P.C. (2011). Assessment of the methodological strategies adopted by food safety training programmes for food service workers: a systematic review. Food Control.

[23] Kyarisiima C.C., Magala H., Kwizera H., Rugira Kugonza D. (2011).

[24] Adedire A.O., Akintunde, Bamiwuye O.A., Aluko O.P., Olutola O.A. (2023).

[25] Ndenga C. (2019).

[26] Namkung Y., Jang S.C. (2007). Does food quality really matter in restaurants? Its impact on customer satisfaction and behavioral intentions. J. Hosp. Tour. Res..

[27] Dabassa A. (2013). Evaluation of home slaughtered meat quality used for human consumption at household and food seller house in Jimma. J. Med. Sci..

[28] Chea R. (2023).

[29] Mostafidi M., Sanjabi M.R., Shirkhan F., Zahedi M.T. (2020). A review of recent trends in the development of the microbial safety of fruits and vegetables. Trends Food Sci. Technol..

[30] Ibrahim H.M., Hassan M.A., Amin R.A., Amin A.K. (2021). Effects of lemon and kiwi juice in reduction of some pathogens contaminating chicken breast meat. Benha Vet Med J.

[31] Gill C.O. (1996). Extending the storage life of raw chilled meats. Meat Sci..

[32] Pradhan A.K., Li M., Li Y., Kelso L.C., Costello T.A., Johnson M.G. (2012). A modified Weibull model for growth and survival of listeria innocua and Salmonella typhimurium in chicken breasts during refrigerated and frozen storage. Poult. Sci..

[33] Girmay A.M., Gari S.R., Gessew G.T., Reta M.T. (2021). Determinants of drinking water quality and sanitary risk levels of water storage in food establishments of Addis ababa, Ethiopia. J. Water Sanitation Hygien. Develop..

[34] Stead M., Angus K., Langley T., Katikireddi S.V., Hinds K., Hilton S. (2019). Mass media to communicate public health messages in six health topic areas: a systematic review and other reviews of the evidence. Public Health Res..

[35] Madjdian D.S., van Asseldonk M., Talsma E.F., Amenu K., Gemeda B.A., Girma S. (2024). Impact of a mass-media consumer awareness campaign on food safety behavior and behavioral determinants among women in Dire Dawa and Harar, Ethiopia. Food Control.

[36] Madjdian D.S., van Asseldonk M., Talsma E.F., Dione M., Ilboudo G., Roesel K. (2024). Empowering consumers to purchase safe ready-to-eat chicken from street restaurants in Ouagadougou, Burkina Faso: impact of a multi-media behavior change campaign. Sci. Rep..

[37] Ovuru K.F., Izah S.C., Ogidi O.I., Imarhiagbe O., Ogwu M.C. (2023). Slaughterhouse facilities in developing nations: sanitation and hygiene practices, microbial contaminants and sustainable management system. Food Sci. Biotechnol..

[38] Kebede M.T., Getu A.A. (2023). Assessment of bacteriological quality and safety of raw meat at slaughterhouse and butchers’ shop (retail outlets) in Assosa town, Beneshangul Gumuz regional state, Western Ethiopia. BMC Microbiol..

[39] Parry-Hanson Kunadu A., Ofosu D.B., Aboagye E., Tano-Debrah K. (2016). Food safety knowledge, attitudes and self-reported practices of food handlers in institutional foodservice in Accra, Ghana. Food Control.

[40] Alimi B.A. (2016). Risk factors in street food practices in developing countries: a review. Food Sci. Human Wellness.

[41] Al Banna M.H., Disu T.R., Kundu S., Ahinkorah B.O., Brazendale K., Seidu A.A. (2021). Factors associated with food safety knowledge and practices among meat handlers in Bangladesh: a cross-sectional study. Environ. Health Prev. Med..

[42] Lema K., Abuhay N., Kindie W., Dagne H., Guadu T. (2020). Food hygiene practice and its determinants among food handlers at university of Gondar, Northwest Ethiopia, 2019. Int J Gen Med.

