# Supplementary material for: Food safety and hygiene practices in the preparation of chicken meat and tomatoes in Harar, Ethiopia: Development of a household food safety index
Source: One Health. 2026 Jul 28;23:101530. doi: 10.1016/j.onehlt.2026.101530 (PMC13452407; doi:10.1016/j.onehlt.2026.101530)
Supplement: Supplementary file 1 [file mmc1.docx]

**Supplementary Material 1: Information sheet and Consent Form**

**Qorannoon faalama foon lukkuu mikiroobisadha irraa dhufu ilaalu - Itoophiyaa**

**qulqulina nyaata Magaalota Afrikaa Keessatti jajabeesu (Push-Pull-Project)**

**Qorannoon kun maallaqaan kan gargaarame UK AID fi Bill and Melinda Gates**

**Foundation irraati**

Akkam jirtu, maqaan koo------------------------------------------------------jedhama. Ani kan

dhufe Yuniversiitii Haramaya irraa yoo ta’u, dhaabata ILRI bakka bu'uun qorannoof foon

lukkuu irratti dalagamu gageessufan dhufe. Kaayyoon qorannoo kanaa yeroo foon lukkuu

mana keessatti qophaa’u hamami akka ini gosa bakteeriyaa (non-typhi Salmonella and

Campylobacter) faalamu beekuudha. Haaluma kana hubachuuf, gochaale idilee isiin yeroo

foon lukku qopheessitan hordofaa ilaaluuf, adeemsa keessan gidduu gidduutti imoo suuraa kaasufii viidiyoo waraabuu dandeenya. Akkasumas saamuda/edattoo garagaraa kanneen akka: foon lukkuu, qodaa foon lukku ittiin qopheessan irraa fudhanee faalama inni

jarmootan/microbial qabu ilaalla.

Marii kana irratti hirmaannaan keessan guutummaatti fedhii keessanii. kanaafu, yeroo kamiyyuu hirmaachuu yoo hin barbaanne dhaabuu dandeessu. Kuusaaleen odeeffannoo qoranoo kana ta’ee galmee barreeffamaa kamiyyuu kooppii kan qabatu qorataa fi qindeessaa qofa. Koppiiwwan dijiitaalaa odeeffannoo kana haala amansiisa qabuun ni kuufama. ragaan dhuunfa hundumtuu kompiitara amansiisa ta’een kan qabamuufi kan kuufamu ta’a.

maqaa_____________________malattoo______________________guyyaa_________

**Odeefanoo Dabalataaf**

| **Name** | **Affiliation** | **Email** | **Postal address** | **Phone** |
| --- | --- | --- | --- | --- |
| Theodore  Knight-Jones | Pull-Push  Project Lead  ILRI | t.knight-jones@cgiar.org | International Livestock  Research Institution, PO  Box 5689, Addis Ababa,  Ethiopia | +251-11 617  2000 |
| Kebede  Amenu | Pull-Push  Project Lead  ILRI | K.Amenu@cgiar.  org | International Livestock  Research Institution, PO  Box 5689, Addis Ababa,  Ethiopia | +251  911087930 |
| Sisay  Girma | Haramaya  University | Sisaygirma80@g  mail.com | P.O. Pox, 138 Dire Dawa  Ethiopia | +25191308790  4 |

**የዶሮ ስጋ ማይከሮቢያል ብክለት ዳሰሳ ጥናት- ኢትዮጵያ**

**በአፍሪካ ውስጥ በከተማ የምግብ ገበያዎች የምግብ ደህንነትን ማበረታታት (Push-Pull-Project)**

**የፑል-ፑሽ ፕሮጀክት ዳራ፡-** የዚህ ጥናት አላማ ከዶሮ እና አትክልት ምግብ ደህንነት ጋር የተያያዙ የተመጋቢዎች የዶሮ ስጋ አዘገጃጀት ልምዶችን በሐረር በተመረጡ ቤተሰቦችላይ ማከናወን::

**የገንዘብ ድጋፍ፡-** ይህ ጥናት በ UK AID እና Bill and Melinda Gates ፋውንዴሽን የገንዘብ ድጋፍ የተደረገ ነው።

**በመረጃ የተደገፈ ስምምነት**

ሠላም ስሜ ______________________

እኔ ከሀሮማያ ዩኒቨርሲቲ እና ዓለም ዓቀፍ የእንስሳት ምርምር ተቋም (ILRI) ወክዬ መጥቻለሁ።

የዚህ ጥናት ዓላማ በሐረር የዶሮ ስጋ በቤት ውስጥ በሚዘጋጅበት ጊዜ የምግብ ደህንነትን እና ብክለትን በተመለከተ ለማወቅ ነው። . ይህንን ለመረዳት የዶሮ ስጋን በምታዘጋጁበት ጊዜ የምትጠቀሙቸውን ልምዶች መከተል እና የሂደቱን ምስሎች ማንሳት እና ቪዲዮዎችን መቅዳት እንችላለን።.

የእርስዎን ተሳትፎ በጣም እናደንቃለን። ምልከታዎቻችን በከተማዎ ውስጥ የምግብ ደህንነትን የበለጠ ለመረዳት እና ለማቀድ ይረዱናል። ለምንጠይቃቸው ጥያቄዎች ምንም የተሳሳቱ የሚባሉ መልሶች የሉም። በዚህ ጥናት ውስጥ በመሳተፍዎ ምክንያት ምንም አይነት አደጋ አይኖርም እና ተሳትፎ ሙሉ በሙሉ በፈቃደኝነት ላይ የተመሰረተ ነው። በማንኛውም ጊዜ እና ያለ ምንም ምክንያት ከጥናቱ መውጣት ይችላሉ።

በማንኛውም ጊዜ የእርስዎን ግላዊነት እና ሚስጥራዊነት ዋስትና እናከብራለን። ያቀረቡት መረጃ እና አድራሻዎች በጥብቅ ሚስጥራዊ ናቸው እና ይህንን ለጥናት ዓላማዎች ብቻ እንጠቀማለን።

ተጨማሪ መረጃ ከፈለጉ ሁል ጊዜ ሊያገኙን ይችላሉ (የመረጃ ወረቀቱን ይመልከቱ)።

በዚህ ጥናት በሁለቱም ጊዜያት ለመሳተፍ ፈቃደኛ ኖት? አዎ………… አይ……….

ይህን ቅጽ በመፈረም ከላይ የተገለጹትን የስምምነት ውሎች እንደተረዱ እና እንደተስማሙ አረጋግጠዋል።

ስም_____________________________ ቀን ____________ ፊርማ __________

| **Name** | **Affiliation** | **Email** | **Postal address** | **Phone** |
| --- | --- | --- | --- | --- |
| Theodore  Knight-Jones | Pull-Push  Project Lead  ILRI | t.knight-jones@cgiar.org | International Livestock  Research Institution, PO  Box 5689, Addis Ababa,  Ethiopia | +251-11 617  2000 |
| Kebede  Amenu | Pull-Push  Project Lead  ILRI | K.Amenu@cgiar.  org | International Livestock  Research Institution, PO  Box 5689, Addis Ababa,  Ethiopia | +251  911087930 |
| Sisay  Girma | Haramaya  University | Sisaygirma80@g  mail.com | P.O. Pox, 138 Dire Dawa  Ethiopia | +25191308790  4 |
