# Supplementary material for: Food safety and hygiene practices in the preparation of chicken meat and tomatoes in Harar, Ethiopia: Development of a household food safety index
Source: One Health. 2026 Jul 28;23:101530. doi: 10.1016/j.onehlt.2026.101530 (PMC13452407; doi:10.1016/j.onehlt.2026.101530)
Supplement: Supplementary file 2 [file mmc2.docx]

Pull-push project- Chicken survey Harar

Start date and time

yyyy-mm-dd	hh:mm

Enumerator name

 Yohannes Mohammed Mina

 Jemal

 Other (specify next)

Name of additional enumerator

Woreda

 Amir Nur Abadir Shenkor Abokar Jen'ela Hakim

Kebele (01-19)

*Put numbers*

Enter householdID

*This is ID is given from before your start*

Has the replacement of the household made?

 Yes No

If yes, provide the reason briefly

Capture GPS coordinates of the household

latitude (x.y °)

longitude (x.y °)

altitude (m)

accuracy (m)

Hello, my name is	I am here from Haramaya University, and on behalf of ILRI. the objective of the survey is

to assess the knowledge, attitude and practices of home preparation of chicken and vegetable (tomato) related to food hygiene and safety. Thus we want to assess how you handle, process and consume chicken meat and vegetables (especially tomatoes) by focusing on preparation practices of chicken meat and tomato related hygiene and potential cross- contamination with implication to food safety and human health.

There will be no influence on whether or not you decide to talk to us about participating in the study. Your participation in the discussion is completely voluntary, so you are free to leave if you no longer wish to participate at any time. The interview will take about 40 minutes. The research team is committed to respecting your privacy and confidentiality. If you decide not to answer, there will be no negative consequences with the research team and partners, now or in the future.

The data collected during this study will be in an electronic data collection form. Databases will also be prepared. Only the researcher and the coordinator will retain copies of any written records. Digital copies of the information will be stored securely. All data and interviewees' personal information (names and contact details) will be stored on secure computers owned by the research team.

Does household consent to this survey ?

*select only one*

 Yes No

# Group 1

Name of the household head

Sex of the household head

 Male Female

What is your name (woman or girl)?

*Confirm that the respondent is largely involved in food preparation at home*

How old are you?

Mobile telephone number

*Use 911087930 format (nine digits)*

What is your highest education level?

*select one*

 No Formal Education

 Basic Informal Literacy i.e Writing And Reading Elementary School

 Secondary Level Education TVET/College

 University

1.1. How many times per week your family eat CHICKEN MEAT (in a normal week, outside of holiday periods) at home or if you eat them every month or year?

*select one*

 1 times a week

 2 times a week

 3 times a week

 4 times a week

 5 times a week

 6 times a week

 7 times a week monthly

 in 6 month yearly

 other

if other specify

1.2. How many times per week your family eat TOMATOES (in a normal week, outside of holiday periods) at home or if you eat them every month or year?

*select one*

 1 times a week

 2 times a week

 3 times a week

 4 times a week

 5 times a week

 6 times a week

 7 times a week monthly

 in 6 month yearly

 other

if other specify

1.3. How many times per week your family eat LETTUCE (in a normal week, outside of holiday periods) at home or if you eat them every month or year?

*select one*

 1 times a week

 2 times a week

 3 times a week

 4 times a week

 5 times a week

 6 times a week

 7 times a week monthly

 in 6 month yearly

 other

if other specify

2. How frequent you perform the

following chicken buying practices and indicate the frequency of each practice by telling me as Commonly, Sometimes or Never.

Buying live chicken and slaughter at home

Buying live chicken and request to be slaughtered at the market

Buying frozen chicken brought from central Ethiopia

Buying frozen dressed chicken brought from Somaliland or Djibouti (cross-border

Slaughter from own backyard chicken farm

Commonly	Sometimes	Never

3. In which of the following cooking forms are chicken meat prepared at home in your household for consumption?

*select that all apply*

 Doro wot (traditional spiced chicken stew) Roasted whole chicken/doro arousto/

 Chicken soup

 Chicken salad (mixed with vegetable) Chicken with rice

 Other/specify/

if other specify

4. When you buy live chicken, among the following practices which best describes related to slaughtering the chicken? [you choose only one]

*selecte one*

 Slaughtered on the same day, without keeping Keep the chicken for a night before slaughtering

 Keep the chicken for two nights before slaughtering

 Keep the chicken three and more than three nights before slaughtering Other (specify)

Other (practice of live chicken)

5. Where specific place you keep live chicken at your home before slaughtering?

 Kitchen (food preparation area) Living room (salon)

 Store

 Outside of the house Other (specify)

Other keeping area (write it)

6. Where is the chicken killed? [tick which applies)

 inside living area (salon)

 inside food preparation area (kitchen) Outisde of the house

 Other (specify)

Other chicken slaughter area

7. Where do you dispose the remains of the chicken after slaughtering?

 In the compound in temporary place Outside the compound

 Other (specify)

Other disposal place

8. Do you ever slaughter sick birds for consumption?

*select one*

 Yes No

8.1. If yes, how often?

 Most often Often

 Sometimes Rarely

9. What is the MOST common water source for food preparation, including washing of kitchen utensils for your family?

Pipe water Well water River water

Harvested rainwater Other (specify)

Other water source

10. If you use pipe water, how often do you experience interruption of water supply?

Most often Often

Sometimes Rare

11. When there are interruptions of piped water supply, what will be your alternative water sources?

*select all that apply*

River

Well water Rain water

Buying pipe water from neighbors Open tank (E.g. Barrel)

Other (specify)

if other specify

12. Does your family have a functional fridge at home?

*select one*

yes no

12.0. Do you share your neighbour fridge or freezer for cooling foods including chicken meat?

Yes No

12.1. If you have fridge, does it have deep freeze on top?

Yes No

13. Is there a separate deep freezer in your family?

*select one*

yes no

14. Do you keep chicken carcass in freezer after slaughtering at home till you cook it?

*select one*

yes no

15. For how many days approximately do you keep chicken carcass in freezer after slaughtering at home till you cook it? [Number days]
16. Do you store for sometime the carcass that you bought from market till you cook it in freezer?

*select one*

Yes No

17. For how many days approximately do you keep the carcass that you bought frozen at home till you cook it? [Number of days]
18. In what form do you keep home-slaughtered chicken in deep freezer?

Whole chicken without washing

Organ partitioned chicken without washing Organ partitioned chicken after washing Other (specify)

Other chicken meat storage in fridge

*Describe it*

# Gruop 2

19. How often do you follow the following practices when preparing chicken meat at home?

Always	Sometimes	Never

Use of lemon for washing chicken carcass

Neck of the chicken severed and bled outside of the kitchen

When chicken is killed, covered with plastic tub/safa/

Use of hot water for scalding

Plucking of large feathers without using hot water

Use of fire to remove small feathers

Removing chicken skin before cooking

Use of soap (for washing chicken preparation utensils)

Use of lemon for soaking chicken meat

Use of onion wash for soaking chicken meat

Use of peas powder/shiro/ to cover chicken carcass before washing

20. How long you soak chicken carcass in either in lemon?

*select one*

<30 minutes

>more than 30 minutes

21. How long you soak chicken carcass in either in onion wash water?

*select one*

<30 minutes

>more than 30 minutes

22. For how many hours do you keep at room temperature a ready to eat chicken meat/dorowot/ before serving?

*/indicate in hours*

23. For how many hours do you keep at room temperature a ready to eat chicken meat stew/dorowot/ leftover after eating a portion of it?

*/indicate in hours*

24. What are the possible problems of chicken slaughtering at home in terms of hygiene (multiple options possible)? [ interviewer: do not reader the answers]

*Do not read the options*

Contaminate the house environment with potentially harmful germs Can contaminate other food items in the kitchen

Create bad odor in house

Can be burden for domestic waste disposal No problem at all

Other (specify)

Other problems

25. Is there an intention to stop slaughtering of live chicken at home in your family?

Yes No

26. If no intention to stop, why?

Chicken home slaughtering has cultural or religious reasons It is not safe to purchase already slaughtered chicken

The quality and taste of chicken slaughtered and dressed not good We do not find slaughtered and dressed on market

Other (specify)

Other reason not stop

27. If there is an intention to stop slaughtering of live chicken at home, what are the possible reasons?

Limited slaughtering space at home Home environment contamination Shortage of time

Afraid of severing the neck of the animal Other (specify)

Other reason to stop

28. Which of the following best describe your common practice in cleaning kitchen utensils (knife, board, table) just BEFORE use?

Wash with water

Washed with detergent (soap) Washed with warm water

29. Which of the following best describe your common practice in cleaning kitchen utensils (knife, board, table) just AFTER use?

Wash with water

Washed with detergent (soap) Washed with warm water

30. How often kitchen working surface is cleaned with detergent after chicken preparation before using it for preparation other foods?

Always Usually

Sometimes Rarely

Never

31. Where do you keep raw chicken meat in the fridge rack compared to the ready to eat food?

On the top rack above the ready to eat food On the bottom rack below the ready to eat food Anywhere (no specific place)

32. How many days it will take on you before you utilize the first tomato from the batch you bought that is typically used for salads?
33. How many days it will take on you before you utilize the first tomato from the batch you bought that is typically used for sauce?
34. How many days it will take until you use the last tomato out of a batch purchased, typically the one that is commonly used for salads?
35. How many days it will take until you use the last tomato out of a batch purchased, typically the one that is commonly used for sauce?
36. Where do you store tomatoes?

*select all that apply*

In home - refrigerator Not refrigerated

Other (specify)

Other tomato storage

37. Do you remove tomatoes that spoil during storage at home?

yes no

38. How many days per week/month do your family eat raw tomato/salad on average?

Never

1 time per week

2 times per week

3 times per week

4 times per week

5 times per week

6 times per week

7 times per week 1 time in 2 weeks 1 time per month

» 39. How often the following tomato-based foods are consumed at your home in normal times (i.e., out of holidays)?

39. How often the following tomato- based foods are consumed at your home in normal times (i.e., out of holidays)?

Tomato sauce

Salad (timatim kurt) Slightlyheated (leb-leb)

Always	Usually	Sometimes	Rarely	Never

40. How long do you keep the folllowing prepared tomato dishes at room temperature before consumption?

Tomato sauce

Salad (timatim kurt) Slightlyheated (leb-leb)

41. How frequently do you perform the

following practices and processes during preparing tomatoes at home? Select a practice and indicate the frequency of each practice by stating it as ALWAYS, Sometimes, or Never.

Washing tomatoes before preparing

Using disinfecting agents or soap (specify which) to wash tomatoes?

Using salt in water to wash tomatoes Using lemon to wash tomatoes

Using lemon or vinegar when served as topping

Using disinfecting agents or soap for washing cutting boards, knives, and other utensils?

Using hot water for washing cutting boards and knives?

Trimming damaged parts of tomatoes before preparing?

Using separate cutting board/knives for intact and extensively damaged tomatoes?

Using separate cutting board and knives for vegetables and chicken?

Using separate cutting board and knives for vegetables and chicken?

Wash hands with soap after each food item preparation?

<=2 hr	3-5 hr	>= 6 hr

Always	Sometimes	Never practicing

42. Which information sources do you use regarding safe handling and preparation of foods?

*select all that apply*

TV

Magazines/news paper Brochures/leaflets

Social media (Facebook, twitter, YouTube) Recipe pages or books

Not using any

Friends or relatives Other(specify)

if other specify

43. What is your household size (number of people living in the same house and sharing meal)?
44. How many people in your family are with age less than 5 years?
45. How many people in your family are with age greater than 65 years?
46. What is the main income source of your family?

Government organization employed

Non-governmental organization employed Trading as main income source

Informal employment (e.g., assisting other families in domestic works) Remittance from another person living somewhere

Other (specify)

Other main income source

47. What types of house is your family living in? [Ask or Observe]

Villa/L-shape type with separate compound (not shared by other family) Villa/L-shape type with shared [compound with other family]

Condominium (communal house) Service corner (rooms)

Other (specify)

Other house type

48. How is your family house ownership?

Own property

Rent from a private Rent from municipality other (specify)

if other ownership specify

49. How many rooms has your house?
50. Do you have separate kitchen for food preparation?

yes no

51. From which of the following assets your family own?

Television Car (vehicle)

Motor bicycles Bajaji

Farm /land

52. Please have a look at this picture. [SHOW PICTURE] At the top of the ladder are the people who are the best off in your society, those who have the most money, most education, and best jobs. At the bottom are the people who are the worst off, those who have the least money, least education, worst jobs, or no job. Please indicate the rung that best represents where you think you stand on the ladder."

53. As I have stated before, we will have further study related to chicken meat preparation at home. Are you interested to be part of that?

Yes No

End date and time

yyyy-mm-dd	hh:mm

Thank you and this is the end
