# Supplementary material for: Food safety and hygiene practices in the preparation of chicken meat and tomatoes in Harar, Ethiopia: Development of a household food safety index
Source: One Health. 2026 Jul 28;23:101530. doi: 10.1016/j.onehlt.2026.101530 (PMC13452407; doi:10.1016/j.onehlt.2026.101530)
Supplement: Supplementary file 3 [file mmc3.docx]

**Supplementary Material 3**

Complete details of statical methods used in the study data analysis

1. **Bivariable investigation**

We first investigated the relation between potential predictors and the outcome (HGHP score).

*1.1 Numerical predictors*

Pearsons’s correlation numerical predictors and HGHP score

| **Variable** | **Correlation** | **p** | **Explanation** |  |
| --- | --- | --- | --- | --- |
| age | 0.0287 | 0.6506 | age of respondent | Expected direction, very small not significant |
| hh_size | 0.0759 | 0.2302 | Nº in households | Probably confounded with wealth, age, education |
| family_5year | 0.0139 | 0.8267 | Nº < 5years age | Not a predictor – assesses vulnerable groups |
| family_65y~r | 0.0078 | 0.9014 | Nº>65 years age | Not a predictor – assesses vulnerable groups |
| Asset fridge | 0.0809 | 0.2007 | Nº assets (except fridge) | Expected direction, small, not sig |
| number_room_missed | -0.0266 | 0.684 | Nº rooms (16 have 0 rooms in error) | Unexpected direction, variable has missing/incorrect data, drop |

None are individually significant but for regression it is can be useful to include predictors if there is evidence that they are a cause of the outcome, or a known confounder (linked to both causes and outcome) or if they improve model fit or help adjust standard errors.

*1.2 Ordinal predictors*

We re-coded so they ascend from hypothesised worst in terms of hygiene to better (e.g. as education, employment status, housing, wealth improves GHP should also improve). We used Pearson’s correlation to look at strength of association. Overall correlations were very weak and often in unexpected directions.

| **Construct** | **Levels** | **GHP score** | **Frequency** | **Keep in model** |
| --- | --- | --- | --- | --- |
| **Education – “none” was high? May be domestic workers. College unexpectedly low?** | none | 24.8 | 35 | Unexpected small negative correlation (-0.1) with GHP and no logical ordinal relation but plausible causal link. Maybe don’t keep? |
|  | informal | 22.5 | 26 |  |
|  | primary | 22.2 | 74 |  |
|  | secondary | 23.8 | 69 |  |
|  | College/TVET | 18.7 | 33 |  |
|  | university | 23.8 | 15 |  |
|  |  |  |  |  |
| **Employment: informal high? May be domestic workers. Govt may outsource** | Informal | 23.1 | 75 | Very small (o.01) positive correlation with GHP but the lowest category had highest score. Literature says better off = better hygiene, but here kitchen proximity may be key   Maybe don’t keep |
|  | Remittance | 23.0 | 6 |  |
|  | Government | 22.6 | 88 |  |
|  | NGO | 21.9 | 8 |  |
|  | Trade | 22.3 | 75 |  |
|  |  |  |  |  |
| **Type house** | Service corner | 23.8 | 98 | Very small (0.02) positive correlation with GHP but no strong pattern. One category very small.  Maybe don’t keep |
|  | Condominium | 9.7 | 3 |  |
|  | Villa shared compound | 22.4 | 58 |  |
|  | Villa separate compound | 22.1 | 93 |  |
|  |  |  |  |  |
|  | Rent privat (incl relative) | 22.3 | 47 | Small positive correlation (0.08) & Increasing as predicted but little difference between categories. Maybe keep |
|  | Rent municipal | 22.6 | 92 |  |
|  | Own property | 22.8 | 112 |  |
|  |  |  |  |  |
| **Self-reported SES** | 1 | 23.6 | 18 | Looks unstable, again the lowest status is unexpectedly high – perhaps includes many domestic workers. Highest is high as predicted but very small categories.  Maybe don’t keep |
|  | 2 | 22.6 | 62 |  |
|  | 3 | 21.4 | 70 |  |
|  | 4 | 20.6 | 38 |  |
|  | 5 | 25.2 | 49 |  |
|  | 6 | 25.1 | 9 |  |
|  | 7 | 22.0 | 3 |  |
|  | 8 | 26.7 | 3 |  |
|  |  |  |  |  |
| **Assets owned out of 8 – TV, *bajaj*, motorbike, car, farm, house (fridge not included as hygiene infrastructure)** | 1 | 20.5 | 59 | One worst but four and five unexpectedly low. Very few in higher categories |
|  | 2 | 22.5 | 26 |  |
|  | 3 | 24.5 | 72 |  |
|  | 4 | 22.5 | 71 |  |
|  | 5 | 22.9 | 21 |  |
|  | 6 | 26.0 | 2 |  |
|  | 7 | 23.0 | 1 |  |

1.3 *Binary predictors*

Unlike numerical and ordinal predictors, several binary predictors had moderate and significant correlations with the outcome.

| **Variable** | **Correlation** | **p-value** | **Include** | **Notes** |
| --- | --- | --- | --- | --- |
| Male | -0.0155 | 0.8062 | Maybe | Expected direction but very small |
| Woreda rating high (good infrastructure | 0.164 | 0.009 | Yes | Moderate but significant; theoretically plausible |
| Separate kitchen | -0.074 | 0.244 | No | Counter-intuitive & not significant |
| Works for govt or NGO | 0.041 | 0.518 | No | Weak, non-sig |
| Owns home | 0.025 | 0.693 | No | Weak already captured elsewhere) |
| Low self-reported SES | 0.037 | 0.562 | No | Weak and contradictory |
| High self-reported SES | 0.144 | 0.023 | Yes | Moderate and significant |
| Individual assets | All < 0.07 | All > 0.05 | No | Weak, inconsistent |
| Has a fridge freezer | 0.12 | 0.058 | Maybe | Near significant; proxy for wealth & kitchen quality |
| Gets food safety info from tv | 0.293 | 0 | Yes | Strongest correlation |
| Food safety info brochure | 0.139 | 0.027 | Yes | Significant |
| Food safety info from health worker | -0.257 | 0 | Yes | Strong and **negative**, important to understand |
| Info from others (paper, social media, book, none) | Very weak | >0.1 | No | Not significant or useful |
| Thinks home slaughter of poultry is fine | 0.476 | 0 | Yes | Strongest positive predictor — definitely include |

2. **Exploration using Directed Acyclic Graph (DAG)**

The Knowledge, Attitude, Practice (KAP) conceptual framework posits that food safety knowledge and food safety attitude lead to food safety practice. Knowledge (K) directly influences practices (P), while also working indirectly through Attitude (A) as a mediator. The COM-B model for behaviour change, adds the important concept of external factors or Opportunity, which captures the external factors that enable good practice. In this case, neighbourhood infrastructure and household wealth are enabling factors.

To assess these relationships, we built a series of multilevel linear regression models to account for clustering by kebele and woreda. Our primary model will include Knowledge, Attitude, and Opportunity as predictors of Practice, adjusting for relevant confounders such as age and sex.

To estimate the direct and indirect effects of Knowledge on Practice, we conducted mediation analysis, first by modelling Practice as a function of both Knowledge and Attitude, and then by modelling Attitude as a function of Knowledge. This sequential modelling approach allows us to decompose the total effect of Knowledge into its direct and mediated components, following standard causal mediation methods.

**Figure 1: Directed Acyclic Graph**

3. **Shortlist of variables for predicting GHP (causes, confounders, mediators)**

We used rules of thumb for selection of choice of variables to us in model

| **Variable** | **Mechanism** | **Bivar** | **DAG** |
| --- | --- | --- | --- |
| Good knowledge | KAP theoretical framework: Good Knowledge leads to good Practice.  Good Knowledge also leads to good Attitude leads to good Practice | ++++ | ++++ |
| Good attitude | Good Attitude causes good Practice but Attitude also a mediator – the KAP framework is: Attitude independently causes Practice, but also good Knowledge leads to good Attitude leads to good Practice | ++++ | ++++ |
| Infrastructure  Woreda rating | COM-B theoretical framework: Causal- primarily enables better Practice but less effect on Knowledge and Attitude (except as correlated to other SE factors) | ++++ | + |
| Demographic: Age | Confounders: exist prior to KAP and not on causal pathway K>A>P, but influence KAP | + | ++ |
| Demographic:  Sex |  | + | + |
| Demographic:  Household size |  | + | + |
| Education | Confounder & cause: Education likely direct **negative** on Practice as educated do less food handling; minor positive cause through resources. Confounder through influence on wealth & Attitude | ++ | +++ |
| Wealth | Confounder & minor cause: wealth helps access to Education & Knowledge; shapes values & priorities (Attitude); also cause provides resources for Practice | ++ | +++ |

*DAG: Strength of causal relation based on logic and literature*

4. **Checking for clustering using intra-cluster correlation coefficient (ICC):**

Households were clustered in kebeles and kebeles in woredas. Sampling was random from the list of households of all woredas (not stratified by woreda or kebele).

The intraclass correlation coefficient (ICC) shows how related items in a group are.

| **ICC Value** | **Interpretation** | **Comment** |
| --- | --- | --- |
| **< 0.05** | Small clustering | Often safe to ignore in large datasets |
| **0.05 – 0.15** | Moderate clustering | Could bias results if ignored |
| **> 0.15** | Strong clustering | Multilevel model strongly advised |
| **> 0.30** | Very strong clustering | Group membership explains large share of variance |

Our ICC for woreda was 0.05 but for kebele was 0.453. This could be because many kebeles were sparsely sampled, a quarter had only one or two households, so of course unusual results are likely by chance. But it might also reflect real differences in kebele characteristics, some may have more active health workers, better sanitation, better off people etc.

5. **Comparing with stepwise model and OLS**

Even though sampling wasn’t stratified, with an ICC of 0.453we have no choice but to account for clustering. We tried two approaches:

- A simple approach where standard OLS is adjusted for clustering using the vce command in stata
- A more complex approach using multi-level modelling (mixed in stata)

The multilevel model does a better job of:

- Providing accurate estimates and standard errors
- Accounting for clustering so we don’t overstate precision
- Reflecting the real-world structure of our data

We compared the mixed model to the simpler one using criteria like AIC and BIC (which measure model fit), and the multilevel model performed better, despite being more complex

6. **Compared to simpler cluster regression model using AIC and BIC**

Key assumption: no single kebele should dominate the model

*regress ghp_pos wor_rating hhmale age hh_nbr_ppl ed_none asst_1 no_problem inf_tv*

*estimates store ols*

*mixed ghp_pos wor_rating hhmale age hh_nbr_ppl ed_none asst_1 no_problem inf_tv|| kebele_in_woreda:*

*estimates store mixed*

*. estimates stats ols mixed*

Akaike's information criterion and Bayesian information criterion

Model |	N	ll(null)	ll(model)	df	AIC	BIC

ols |	252	-900.6929	-848.1943	9	1714.389	1746.153

mixed |	252	. -821.653	11	1665.306	1704.13

*Note: BIC uses N = number of observations. See [R] BIC note.*

AIC Akaike Information Criterion lower is better fit

BIC Bayesian information Criterion penalises complexity more heavily than AIC

Log likelihood measures how well the model fits the data. Higher (i.e. less negative) values indicate better fit.

We compared the multilevel mixed-effects model with a simpler linear regression model adjusted for kebele-level clustering. The mixed model had a higher log-likelihood (−821.7 vs −848.2), and lower AIC (1665.3 vs 1714.4) and BIC (1704.1 vs 1746.2), indicating superior overall fit. These differences support the inclusion of random intercepts to account for significant between-kebele variation in hygiene scores. While both models yielded consistent core findings, the mixed model better reflects the data structure and was retained as the preferred specification.

7. **Building multi-level model**

Households were clustered in kebeles and kebeles in woredas.

Multi-level modelling (using mixed command) – we can do two level modelling as woreda is not an important cluster

mixed ghp_pos wor_rating hhmale age hh_nbr_ppl ed_none asst_1 no_problem inf_tv|| kebele_in_woreda:

Performing EM optimization ...

| Mixed-effects ML regression | Number of obs | 252 |
| --- | --- | --- |
| Group variable: kebele_in_wo~a | Number of groups | 18 |
|  | Obs per group: |  |
|  | min | 1 |
|  | avg | 14 |
|  | max | 25 |
|  | Wald chi2(8) | 78.93 |
| Log likelihood = -818.55584 | Prob > chi2 | 0 |

| **Predictors** | **Coefficient** | **Std. err.** | **z** | **P>z** | **[95% conf.**  **interval]** |
| --- | --- | --- | --- | --- | --- |
| Woreda infrastructure rated "good" | 5.22 | 2.29 | 2.28 | 0.023 | 0.73- 9.71 |
| Male-headed household | -0.32 | 0.90 | -0.36 | 0.720 | -2.09 - 1.44 |
| Age of respondent | -0.02 | 0.03 | -0.69 | 0.491 | -0.09 - 0.04 |
| Number of people in household | 0.47 | 0.22 | 2.11 | 0.035 | 0.03 - 0.91 |
| No education | 2.71 | 1.13 | 2.41 | 0.016 | 0.50 - 4.92 |
| Asset (at least one) | -1.26 | 0.91 | -1.38 | 0.169 | -3.04 - 0.53 |
| No problem with home slaughtering | 4.23 | 0.91 | 4.63 | 0.000 | 2.44 - 6.02 |
| Gets food safety information from TV | 4.53 | 0.92 | 4.93 | 0.000 | 2.73 - 6.33 |
| Constant | 12.37 | 2.84 | 4.35 | 0.000 | 6.80 - 17.94 |

Random intercept for kebele nested within woreda (kebele_in_woreda) N = 252, Groups = 18

Observation per group: Min=1, Avg= 14, Max= 25

Random Effects= Intercept (kebele_in_woreda): Var = 0.112,

Model Statistics= Log Likelihood = -818.55584, suggesting that the mixed-effects specification effectively captured the hierarchical structure of the data.

Wald χ² (8) = 78.93, The model demonstrated a good overall fit, Prob > χ² = 0.000

Random-effects parameters	Estimate	Std. err.	[95% conf. interval]

kebele_in_~a: Identity

var(_cons)	22.27403	8.925059	10.15612 - 48.85058

var (Residual)	33.2966	3.07869	27.77766 - 39.91207

LR test vs. linear model: chibar2(01) = 59.28	Prob >= chibar2 = 0.0000

v**ar(_cons)**: This is the **between-kebele variance**, how much kebeles differ in their average hygiene score. That is the estimated variance in the intercepts (i.e. average hygiene scores) between kebeles.

**Residual variance**: The **within-kebele variance**, how much households differ within the same kebele.

LR test vs linear: This compares multilevel model to a regular regression.

- Null hypothesis: No variation between kebeles.
- Result: Strong evidence that multilevel model fits significantly better → multilevel modelling is justified.

Interpretation

| **Variable** | **Framework Role** | **Interpretation** | **Explanation** |
| --- | --- | --- | --- |
| inf_tv (Gets FS knowledge from TV) | Cause (KAP + COM-B) | Expected – strongly associated with better practice | People who acquire Food safety Knowledge, have better Practice. Also reflects active information-seeking, a personal motivation dimension. |
| no_problem with backyard slaughter | Attitude → Practice (Mediator) + independent cause | Expected – strong predictor of good practice | People who care about hygiene prefer the much cleaner backyard slaughter positive Attitude, which leads to better Practice. Strongly supports the Attitude–Practice link in KAP. |
| woreda_rating | Opportunity (COM-B) | Expected but reduced in mixed model | Affects Practice via enabling environment. Significance weakens in multilevel model as kebele random intercept absorbs between-area variance. |
| hhmale, age | Demographic Confounders | Not significant – no strong influence in this context | Hygiene practices may be cultural, role-based, or habitual, not easily modified by these demographic features. |
| hh_nbr_ppl | Proxy/confounder (unclear causal role) | Unexpected – positively associated with better practice | May reflect intra-household division of food roles (larger households more likely to include someone with better practice) or unmeasured social dynamics. |
| ed_none | Confounder & Cause | Unexpected direction – lack of formal education linked to better practice | Likely reflects traditional roles: less educated individuals more likely to cook, thus more practiced and attentive to hygiene. Supports context-specific causal logic. |
| asst_1 | Confounder & minor cause | Expected direction but not significant | Poorer households expected to have worse practices, but other factors (e.g. motivation, exposure, role) may override asset constraints. |

- Description of codes used for analysis

| Code | Description |
| --- | --- |
| hh_id | Household identification |
| Woreda | District |
| num_room_miss | number rooms with 16 missing |
| Kebele (01-19) | Sub-dsitrct |
| mode_impute | number of rooms with missing imputed |
| wor_rating | woreda rated as good infrastructure |
| hhmale | household head male |
| ass_x_fridge | assets not including fridge |
| ed_ord | education none, informal, primary, secondary, college, university |
| ed_sec_up_bi | secondary and bove ed |
| income_source | works for govt, NGO, trade or informal |
| salaried | works for govt or NGO |
| rich_bi | works for govt or business |
| ladder_representation | self-reported ses |
| house_type | house shared villa, own villa, service or condo |
| house_ownership | house own, rented priv, rent munic, belongs relative |
| separate_kitchen | kitchen separate from house |
| no_problem | thinks no problem with backyard slaughter |
| fridge_freeze | owns fridge with freezer compartment |
| inf_tv | gets food safety information from tv |
| inf_ppr | gets food safety information from newspaper |
| inf_broch | gets food safety information from brochures |
| inf_socmed | gets food safety information from social media |
| inf_book | gets food safety information from recipe book |
| inf_none | gets food safety information from nowhere |
| inf_oth | gets food safety information from other source |
| inf_frdrel | gets food safety information from friend/relative |
| ghp_pos | Good Hygienic Practice Index which summarises hygienic behaviour |

8. **Model diagnostics**

We assessed the assumptions underlying a multilevel linear regression model predicting hygienic food handling practices (ghp_pos) in households nested within kebeles. The model includes fixed effects for predictors such as Knowledge, Attitude, and Opportunity (COM-B/KAP framework), and random intercepts at the kebele level to account for within-area clustering. Model diagnostics supported the appropriateness of the multilevel specification, confirming the validity of key assumptions including linearity, normality, homoscedasticity, and independence of residuals.

8.1. **Linearity: each predictor has a linear relationship with the outcome**

We assessed linearity using LOWESS overlays and partial residual plots for two continuous predictors: age and hh_nbr_ppl. Age showed an approximately linear relationship with the outcome. Household size showed minor deviations from linearity in the LOWESS plot, but appeared linear in partial residual plots.

We evaluated transformations using ladder, which suggested a square root transformation for hh_nbr_ppl. However, using the transformed variable produced negligible improvement in model fit (ΔLogL = 0.189), so we retained the original scale for interpretability.

Tests:

- LOWESS with overlying linear regression line for comparison
- partial residual plots (also called component-plus-residual plots)

**LOWESS**

twoway (scatter ghp_pos age) (lfit ghp_pos age) (lowess ghp_pos age)

. twoway (scatter ghp_pos hh_nbr_ppl) (lfit ghp_pos hh_nbr_ppl) (lowess ghp_pos hh_nbr_ppl)

**Interpretation**. If the LOWESS curve hugs the linear line → linearity assumption is ok

**Our result**: Pretty good for age, less good for people in household

**Conclusion**. mild non-linearity for people in hh

**PARTIAL RESIDUAL PLOTS**

regress ghp_pos inf_tv no_problem wor_rating hhmale age hh_nbr_ppl ed_none asst_1

**Interpretation**. Good if the plot looks roughly linear and centered around the line.

**Our result**: Looks good

**Conclusion**. OK

8.2. **Normality of Residuals**

Histograms, Q-Q plots, and a Shapiro–Wilk test (W = 0.990, p = 0.098) indicated that residuals were approximately normally distributed. Deviations in the Q-Q plot tails were not considered serious given the sample size (n = 252). Therefore, the normality assumption was deemed acceptable.

Key assumption: residuals (difference between GHP score and what model predicts) and random effects are approximately normally distributed. Note: the GHP score does NOT have to be approximately normal (it does have to be approximately continuous e.g. behave like numbers not categories).

Think of residuals as “How good or bad was the model at predicting the GHP score for each household?)

Tests

- Histogram of residuals
- Q-Q plot residuals
- Shapiro-Wilk

A **Q-Q plot** “quantile-quantile” plot assesses if residuals in a regression analysis are normally distributed. If residuals fall along a 45-degree straight line they are approximately normal. If they severely deviate, they are not normal and may need transformation

. predict resid, resid

. histogram resid, normal

(bin=15, start=-15.857434, width=1.9839215)

. qnorm resid

**Interpretation**: histogram variables look okay; QQ plot deviations at ends (fat or thin tails)

**Conclusion**. Deviations from normal but is that okay given small number observations.

. predict res_std, rstandard

. . swilk res_std

Shapiro–Wilk W test for normal data

Variable |	Obs	W	V	z	Prob>z

-------------+------------------------------------------------------

res_std |	252	0.99045	1.743	1.294	0.09787

**Interpretation**: Shapiro Wilk p>0.05 so a

**Conclusion**: residuals do not differ from normal

8.3. **Homoscedasticity**

A scatterplot of residuals against fitted values showed no systematic pattern or “fanning” effect, indicating constant variance across the range of predictions. This supports the assumption of homoskedasticity.

Key assumption residuals are equally spread across all predicted values. When a model is well-specified, you'd expect:The accuracy of the model to be roughly the same across all types of predicted values, low, medium, or high. So the size of the errors should not vary with the GHP score. If it does you are missing something. The model is under-specified. If the model is good for poor hygiene people but bad for high hygiene people, the residuals would fan out as you move along the x axis forming a cone. That’s heteroskedasticity

predict resid_mixed, residuals

. predict fitted_mixed, fitted

scatter resid_mixed fitted_mixed, yline(0)

If residuals are evenly scattered around zero, they’re good.

If they fan out (like a cone) or form a curve, that’s heteroskedasticity.

**Interpretation**: looks okay

**Conclusion**: residuals do not differ from normal

8.4. **Normality of random effects**

We plotted the predicted kebele-level random effects. The histogram was slightly non-normal and Q-Q plots showed modest tail deviations. These were attributed to small cluster sizes in several kebeles (e.g., kebeles with 1–2 households). Given that multilevel models apply shrinkage to small clusters, and sensitivity analyses showed no impact on overall model estimates, we retained all kebeles.

Key assumption: random effects are approximately normally distributed.

Kebele random intercepts are the model-estimated deviations in hygiene scores for each kebele from the overall mean

Kebele random effects are the difference between each kebeles intercept and the overall intercept

We assume kebele-level random effects are normally distributed and centred around zero

Think of random effects as “"How much higher or lower is this kebele’s average hygiene compared to the national average?”

If the random effects are very skewed this suggests the kebeles are coming from different populations or the model is missing important variables

. predict kebele_re, reffects

. histogram kebele_re, normal

(bin=15, start=-8.4298105, width=1.0187588)

. qnorm kebele_re

The distribution is less humped and more bimodal, and the q-q plot shows deviation especially at the ends. Some kebele means are more extreme than expected. This could be because some are small in size and so estimates are unreliable, or because some are unusually good or unusually bad, or they are drawn from very different populations (urban, rural, etc).

In our case it is almost certainly because some kebeles are very small, five have only one or two households. We don’ think these kebeles are fundamentally different, they were just sparsely sampled and so got extreme results by chance. Mixed models are robust and automatically shrink small group estimates (Empirical Bayes shrinkage).

We observed some non-normality in the distribution of random effects, likely due to a few small kebeles with very few households. However, since mixed-effects models naturally account for group size through shrinkage, and sensitivity checks confirmed that their inclusion did not meaningfully affect the results, we opted to retain them in the analysis.

8.5. **Independence of residuals**

Residuals were plotted by kebele (boxplots) and against fitted values (scatterplot with kebele color coding). Residuals were generally centered around zero within clusters and showed no systematic within- or between-kebele patterns. This supports the assumption of residual independence, conditional on the random effects.

In a multilevel/mixed model:

- You assume independence of residuals *within* clusters after accounting for the random effects.
- That is, conditional on the random intercept, residuals within a cluster should be uncorrelated.
- Across clusters (e.g., between kebeles), residuals are assumed independent.

Tests

- Check residuals within and across clusters
- Plot residuals against predicted values, cluster by color

graph box resid, over(kebele_in_woreda)

Wide or skewed residual spread in certain kebeles → might indicate unmodeled structure.

**Interpretation**: Residuals appear to be cantered around zero in most kebeles.

The spread (interquartile range) is reasonably consistent across kebeles.

A few kebeles have slightly wider or narrower boxes, but there are no extreme outliers or skewness patterns that suggest major misspecification.

No kebele has all residuals strongly positive or negative.

**Conclusion:** suggests that, after accounting for random intercepts, the residuals within kebeles are approximately cantered and show no obvious structure**.**

*twoway scatter resid fitted_value, mcolor(kebele_in_woreda)*

**Interpretation**: The residuals are evenly scattered around zero, with no visible funnel shape, curve, or clustering along the x-axis (fitted values).

**Conclusion**: Okay

8.6. **Multicollinearity**

Multicollinearity was assessed using Variance Inflation Factors (VIFs) from an OLS model. All VIFs were <1.4, indicating no problematic multicollinearity among predictors.

VIF won’t run after mixed command so run after standard reg

regress ghp_pos wor_rating hhmale age hh_nbr_ppl ed_none asst_1 no_problem inf_tv

. vif

Variable |	VIF	1/VIF

hhmale |	1.34	0.745572

age |	1.25	0.802095

hh_nbr_ppl |	1.12	0.890721

asst_1 |	1.07	0.933489

inf_tv |	1.04	0.959835

no_problem |	1.04	0.966161

ed_none |	1.03	0.969610

wor_rating |	1.01	0.991230

Mean VIF |	1.11

1== no correlation

One to five – moderate correlation – can ignore

>5 potentially severe correlation – requires adjustment

8.7. **Plot fitted (what the model predicts for each household) vs observed (the actual GHP score)**

looking for a tight cloud around the 45° line. Wide scatter suggests the model misses some important predictors or structure. Systematic patterns (e.g., curves) suggest **misspecification**. Large vertical spread = low precision.

Fitted= xB +zU

Where xB is the fixed effects (like attitude, information sources, education) and Zu is the contributiion of random intercepts (in this cae the kebele level adjustment)

So fitted value is what the model predicts ADJUSTED by the kebele its in

. predict fitted, fitted

. scatter ghp_pos fitted

A reasonable relation between predicted and observed. No curves, gaps, stripes. Some spread but this is typical especially given our sampling strategy was not ideal.
