# Supplementary material for: Food safety and hygiene practices in the preparation of chicken meat and tomatoes in Harar, Ethiopia: Development of a household food safety index
Source: One Health. 2026 Jul 28;23:101530. doi: 10.1016/j.onehlt.2026.101530 (PMC13452407; doi:10.1016/j.onehlt.2026.101530)
Supplement: Supplementary file 4 [file mmc4.docx]

| **Supplementary material 4: Lists of variables for chicken and tomato household food safety and construction of hygiene practice index Construction of the HGFHP Index** | | | | | | |
| --- | --- | --- | --- | --- | --- | --- |
| **No.** | **Practice category** | **Variable** | **Description** | **Risk Pathway** | **Coding*** | **Justification** |
| 1 | **Chicken.**  Slaughter | Chicken slaughter days | Days chicken kept at home before slaughter/ same day | Environmental contamination | 1= same day | bad practices since there is no designated area to keep chicken |
| 2 |  | Area where chicken kept before slaughter | Place where chickens are kept before slaughter outside the living room & kitchen | Environmental contamination | 1= Outside | Direct transmission of pathogen from animal to the environment |
| 3 |  | Area where chicken killed | Area where chicken killed outside the living room & kitchen is safe | Environmental contamination | 1= Outside the living room | Exposure to pathogen due to blood spill over |
| 4 |  | Slaughter sick birds | Slaughter of sick birds is risky | Pathogen introduction | 1=No | Higher pathogen load |
| 5 |  | Bled outside | Bleeding location outside | Environmental contamination | 1=Always | Open bleeding spreads pathogens |
| 6 |  | Carcass covered (Slaughtered chicken covered) | Immediately after slaughter the chicken covered by *safa* (big bowl) | Environmental contamination | 1=Yes | Prevents blood spillover and contamination |
| 7 |  | Wash Carcass preparation equipment with soap | Wash Carcass preparation equipment with soap | Cross-contamination | 1=Soap | WHO principle |
| 8 |  | Scalding | The use of hot water after slaughter to remove the feathers always | Surface contamination | 1=Always | Reduce external pathogens using thermal reduction |
| 9 |  | Dispose remains | Chicken waste disposal outside the compound | Environmental contamination | 1=outside | Prevents vector spread |
| 10 | Preparation | Wash carcass with lemon | Carcass wash with lemon always | Microbial reduction | 1=Always | Partial antimicrobial effect |
| 11 |  | Lemon soak | Soaking meat with lemon always | Microbial reduction | Always = 1 | Traditional practice |
| 12 |  | Pea powder scrubbing of the carcass | Scrubbing carcass with pea powder (has paper and salt in it) | Microbial and debris reduction | Yes =1 | Traditional practice |
| 13 |  | Sanitize length | How long the chicken carcass soak in lemon | Microbial reduction | >30min =1 | The longer the soaking time the lemon will have time to kill the bacteria |
| 14 |  | Preparation type | Chicken meal preparation with salad/vegetables | Cross-contamination | -1 = Cooked with salad | Exposure dependent |
| 15 | Post-prep | Time from cooking to serve | Time from cooking to serving 2h/Holding time.  *Removed due to missing data* | Time-temp abuse | ≤2h=1 | WHO guidance |
| 16 |  | Time leftover kept 2hrs or less | The time keep the leftover before consumption ≤2h/Storage duration | Pathogen growth | ≤2h=1 | Critical control |
| 17 | Cold Storage | Keep raw meat in fridge | After washing the chicken carcass refrigerated until usage/ Refrigeration | Bacterial growth | 1=Yes | Reduces growth |
| 18 |  | place of raw meat in bottom fridge | Placement of meat at the bottom of the fridge | Cross-contamination | 1=bottom rack | Prevents drip contamination |
| 19 |  | Keep freeze | keep chicken carcass in freezer after slaughtering / Freezing | Bacteria growth control | 1=Yes | Slows growth |
| 20 |  | Zero day kept in freezer | Days kept in freezer zero | Growth prevention | 1= Yes | Best practice |
| 21 | Water | Alternative water good only barrel | When there is water interruption the type of water used /Alternative water safe | Water contamination | 1=Safe water | Unsafe water risk |
| 22 | Utensil/ surface Cleaning | Utensil clean before use | Utensil clean before use with soap | Cross-contamination | 1=Soap | Core hygiene |
| 23 |  | Utensil clean after use | Utensil clean after use with soap | Residual contamination | 1=Soap | Prevents carryover |
| 24 |  | Surface clean after | Surface clean after always | Cross-contamination | 1=Yes | Critical control |
| 25 | **Tomato;**  Preparation | Wash tomatoes before preparation | Wash tomatoes before preparation always | Contamination removal | 1= Always | Removes pathogens |
| 26 |  | Clean tomato with soap | Wash tomato with soap always | microbial removal | 1=Always | Improved cleaning |
| 27 |  | Trim damaged tomato | Trim damaged tomato always | Pathogen reduction | 1=Always | Spoiled areas risky |
| 28 |  | Separate intact & damaged | Sorting/ Separate intact/damaged tomato always | Spread prevention | 1=Always | Prevents spread |
| 29 |  | Separate veg & chicken | Separate veg & chicken cutting board always | Cross-contamination | 1= Always | Critical control |
| 30 |  | Tomato sanitizer | Use of sanitizer for tomato always | Pathogen reduction | 1=Always | Strong intervention |
| 31 |  | Wash hands after preparation | Wash hands after preparation always | Direct contamination | 1=Always | WHO principle |
| 32 |  | Raw recipes | Raw tomato consumption never | Direct exposure | 1=Never | High risk |
| 33 |  | Lightly cooked recipes | Undercooking tomato consumption | Pathogen survival | 1=Never | Risk remains |
| 34 | Post-preparation | Keep prepare sauce | Keep prepared sauce <2hr | Growth risk | 1= <2hr | Time-temp dependent. |
| 35 |  | Keep prepared salad | Keep prepared salad <2hr | Contamination risk | 1= <2hr | Raw foods risk |
| 36 |  | Keep prepared *leb-leb* | Keep prepared undercooked <2hr | Growth risk | 1= <2hr | Risk remains |
| 37 | Utensils & surface Cleaning | Using disinfectant agent cutting board | Use disinfectant agent cutting board always / Chemical sanitation | Pathogen reduction | 1=Always | High impact |
| 38 |  | Hot water washing for board & knife | washing board & knife using hot water always | Pathogen reduction | 1=Always | Effective |
| 39 | Storage | Remove spoiled | Remove spoiled tomato | Contamination risk | 1=Yes | Good practice to reduce cross contamination |
| 40 |  | Storage place | Tomato storage in fridge | Minimize tomato spoilage | 1= Fridge | Although tomatoes may taste better outside fridge they keep better in fridge |
| 41 |  | Start use for salad | Days it will take before utilizing the first tomato for salad less than a day | Contamination risk | 1=<1 day | Good to use fresh tomato for salad to minimize risk |
| 42 |  | Start use for sauce | Days it will take before utilizing the first tomato for sauce less than a day | Contamination risk | 1=< 1 day | Good to have a practice to use fresh tomato to minimize risk |
| 43 |  | Last use for salad | Days it will take to use the last tomato out of a batch for salad | Contamination risk | 1=≤ 2 days | High impact |
| 44 |  | Last use for sauce | Days it will take to use the last tomato out of a batch for souse | Contamination risk | 1= ≤7 days | Good to have a practice to use fresh tomato to minimize risk |

*The coding column includes only household practices categorized as “good practices” (coded as 1); risky and neutral practices are not shown.
